# Addendum: Sea level much higher than assumed in most coastal hazard assessments

**DOI:** 10.1038/s41586-026-11017-1

**Published:** 2026-08-25

**Authors:** Katharina Seeger, Philip S. J. Minderhoud

**Affiliations:** 1 Soil Geography and Landscape Group, Wageningen University and Research, Wageningen, The Netherlands; 2 Institute of Geography, University of Cologne, Cologne, Germany; 3 Department of Civil, Environmental and Architectural Engineering, University of Padova, Padova, Italy; 4 Department of Groundwater and Water Security, Deltares Research Institute, Utrecht, The Netherlands

**Keywords:** Natural hazards, Physical oceanography, Climate-change impacts

Addendum to: *Nature* https://doi.org/10.1038/s41586-026-10196-1 Published online 4 March 2026

## Main

This Addendum provides the configuration details for the geoid models used in the methodology underlying the coastal exposure meta-analysis in the original article^1^. It expands upon the description in the original publication to ensure full reproducibility of our results. The specific configuration details are outlined below and in Extended Data Tables 1–5.

Geoid model information was obtained from the openly accessible calculation service of the International Centre for Global Earth Models from GFZ Helmholtz Centre for Geosciences (ICGEM)^2^. Point data on geoid height anomaly to the WGS84 ellipsoid (in m) was obtained for the entire globe at a resolution of 0.085° for the EGM96 (ref. ^3^), EGM2008 (ref. ^4^) and GOCO06s (ref. ^5^) geoid models, respectively^6^ (data downloaded on 22/08/2023 for EGM96 and EGM2008 and on 10/01/2024 for GOCO06s) (Extended Data Tables 1–3). Specifically, the output grid from ICGEM documents the maximum latitudes of 90.000 °N and 89.945 °S (2118 latitude parallels), and the maximum longitudes of 180 °W and 179.975 °E (4236 longitude parallels). In total, 8,971,848 points were downloaded for each geoid model. We included the zero-degree term as given by ICGEM into the geoid model configuration^7^ and for each geoid model refer to a tide-free system configuration^6^. The reference ellipsoid WGS84 underlying these geoid models is defined by the geocentric gravitational constant *GM* = 3.98600441800 × 10^14^ m^3^/s^2^, a radius of *a* = 6378137.00 m, a flattening of *f* = 1/298.25722356300, an angular velocity of ω = 7.292115 × 10^–5^ s^–1^ and a normal potential of *U*_0_ = 6.263685171456948 × 10^7^ m^2^/s^2^. We did not apply any filters in the configuration^6^. Consequently, the geoid model data we obtained from the ICGEM calculation service include the EGM96 (with a maximum used degree of 360) characterized by a weighted mean of –7.1416531 × 10^–3^ m, a maximum of 8.5891824 × 10^1^ m, a minimum of –1.0651816 × 10^2^ m and a weighted root mean square of 3.0566520 × 10^1^ m (Extended Data Table 1); EGM2008 (with a maximum used degree of 2190) characterized by a weighted mean of –8.2435599 × 10^–3^ m, a maximum of 8.6064395 × 10^1^ m, a minimum of –1.0648962 × 10^2^ m and a weighted root mean square of 3.0570532 × 10^1^ m (Extended Data Table 2); and GOCO06s (with a maximum used degree of 300) characterized by a weighted mean of –7.1516965 × 10^–3^ m, a maximum of 8.5419839 × 10^1^ m, a minimum of –1.0651832 × 10^2^ m and a weighted root mean square of 3.0569639 × 10^1^ m (Extended Data Table 3).

For each geoid model, the height anomaly points were interpolated into a global raster by using multiquadric radial basis functions, which gave the most accurate interpolation results and is also used by gravitational field modelling studies^8,9^. The interpolations were conducted using the geostatistical wizard in ArcGIS Pro (version 3.1.3). For each interpolation, the kernel parameter was optimized. The standard neighbourhood type was automatically set with a minimum of 10 and a maximum of 15 neighbours. A sector type of one sector was used.

The processing was similar for our additional computations, involving the GOCO2025s (ref. ^10^) and EGM-DIR R4 (refs. ^11,12^) investigations, which were added in the later process of our article (Extended Data Tables 4 and 5). For both the GOCO2025s and the DIR R4 geoid models, we downloaded height anomaly data to the WGS84 ellipsoid as specified above^6^ (date of download for both geoid models: 03/10/2025). We used a grid step of 1.000° and a global coverage, extending from 90.000 °N to 90.000 °S and from 180.000 °W to 180.000 °E, thus obtaining 65,341 grid points. As for the previously described geoid models, we included the zero-degree term as given by ICGEM into the geoid model configuration^7^ and for each geoid model refer to a tide-free system configuration^6^. Consequently, the output configurated by ICGEM include the GOCO2025s (with a maximum used degree of 300) characterized by a weighted mean of 1.6370365 × 10^–2^ m, a maximum of 8.4607608 × 10^1^ m, a minimum of –1.0634983 × 10^2^ m and a weighted root mean square of 3.0565538 × 10^1^ m (Extended Data Table 4); and the DIR R4 (with a maximum used degree of 260) characterized by a weighted mean of 1.6487188 × 10^–2^ m, a maximum of 8.4596036 × 10^1^ m, a minimum of –1.0649776 × 10^2^ m and a weighted root mean square of 3.0565126 × 10^1^ m (Extended Data Table 5). The respective geoid model point data were interpolated into a global raster using multiquadric radial basis functions with kernel parameter optimization, standard neighbourhood type with minimum 10 and maximum 15 neighbours, and a sector type of one sector. Subsequently, coastal offsets to EGM96 and GOCO06s geoid models and mean dynamic topography (Extended Data Fig. 9 of ref. ^1^; Supplementary Fig. 9 of ref. ^1^) were determined by subtraction and as detailed in Extended Data Fig. 2 of ref. ^1^, followed by the extraction of point values at a 90 m interval along the coastline using bilinear interpolation of values at point locations and excluding no data values and water bodies before rasterization. For visualization purposes, the spatial scale of the data shown in Extended Data Fig. 9 of ref. ^1^ and Supplementary Fig. 9 of ref. ^1^ was resampled to 1° using bilinear resampling, whereas statistics are given at 1,000 m spatial resolution (Supplementary Data 2 of ref. ^1^).

## Figures and Tables

**Extended Data Table 1:**
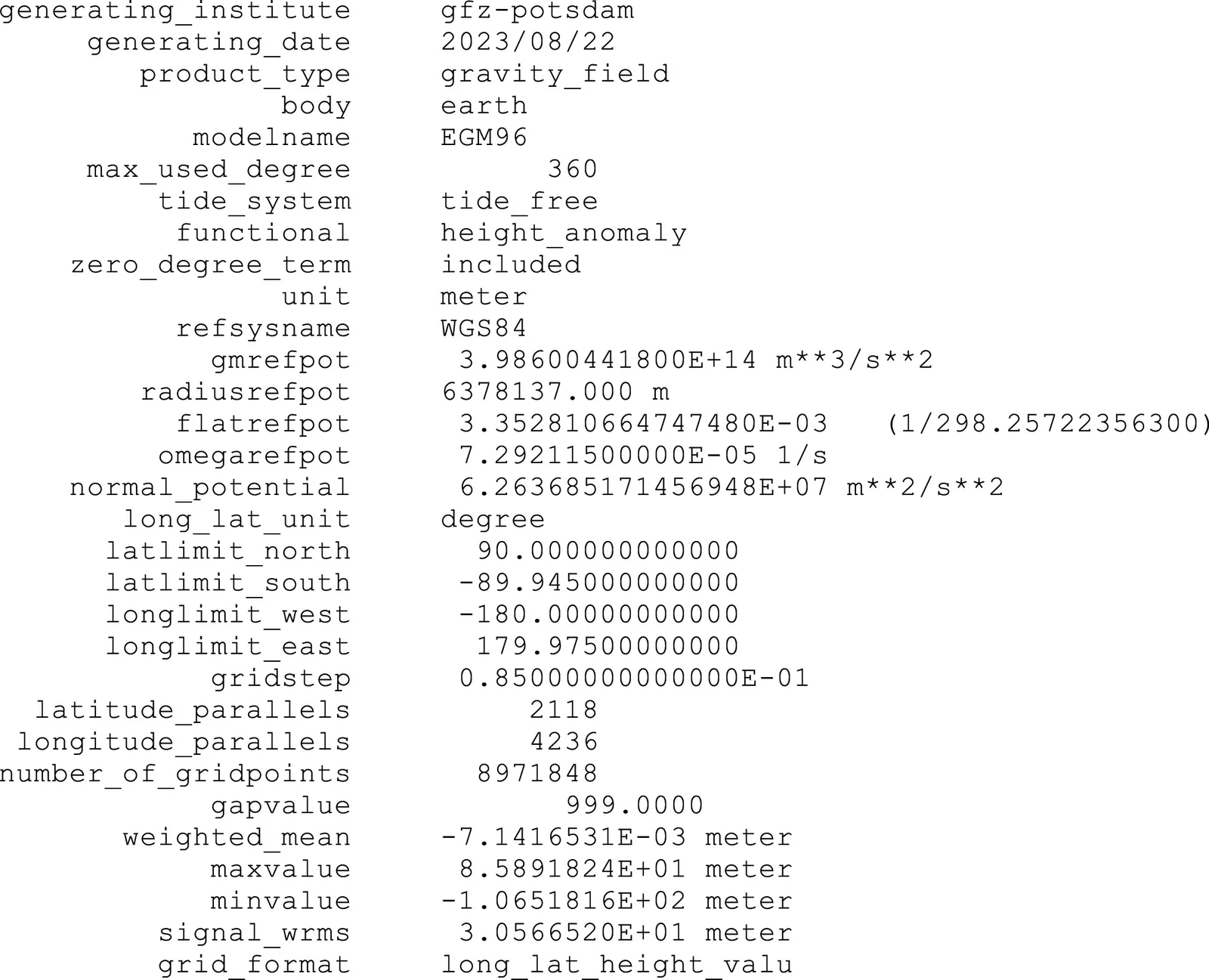
EGM96 model configuration metadata

**Extended Data Table 2:**
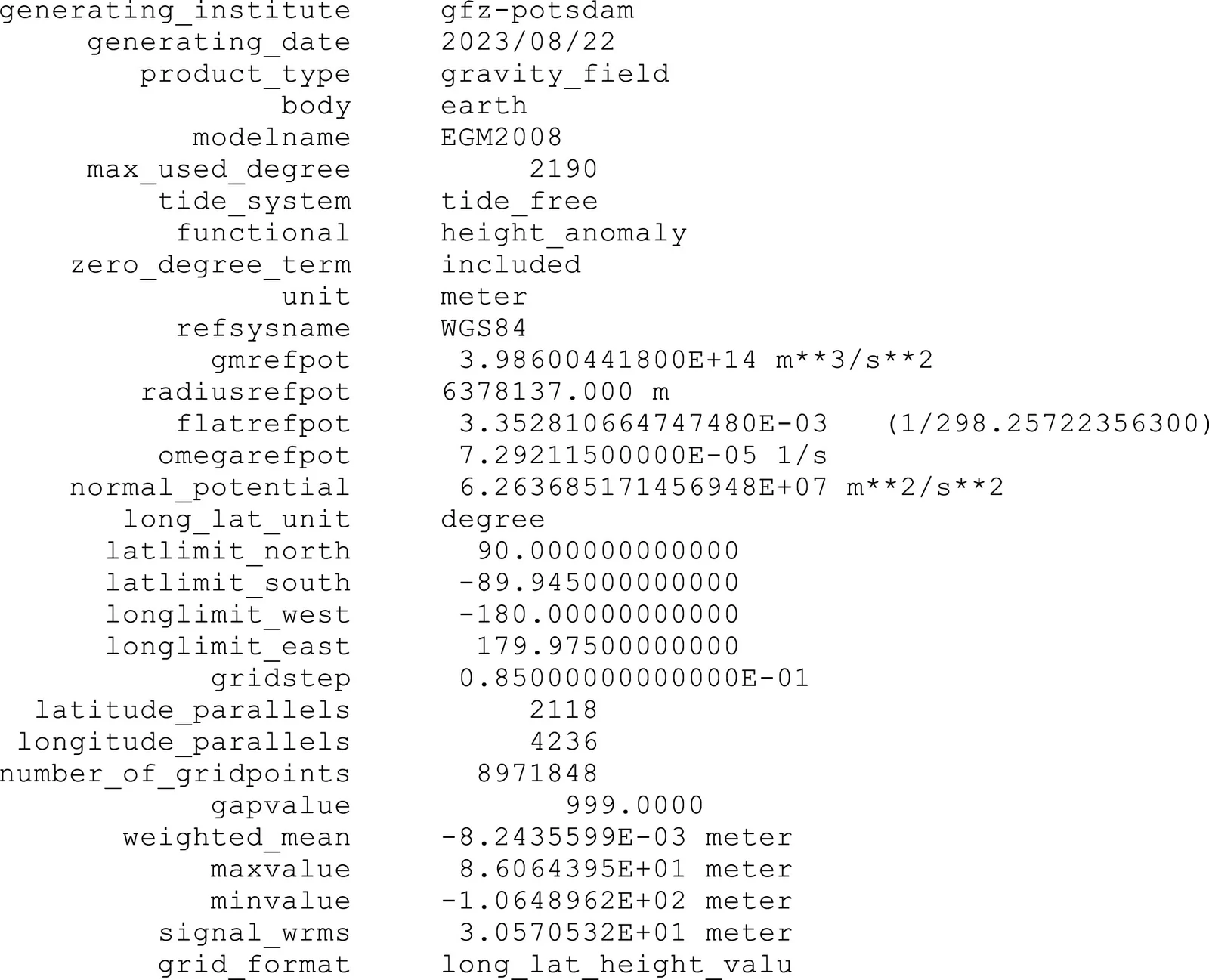
EGM2008 model configuration metadata

**Extended Data Table 3:**
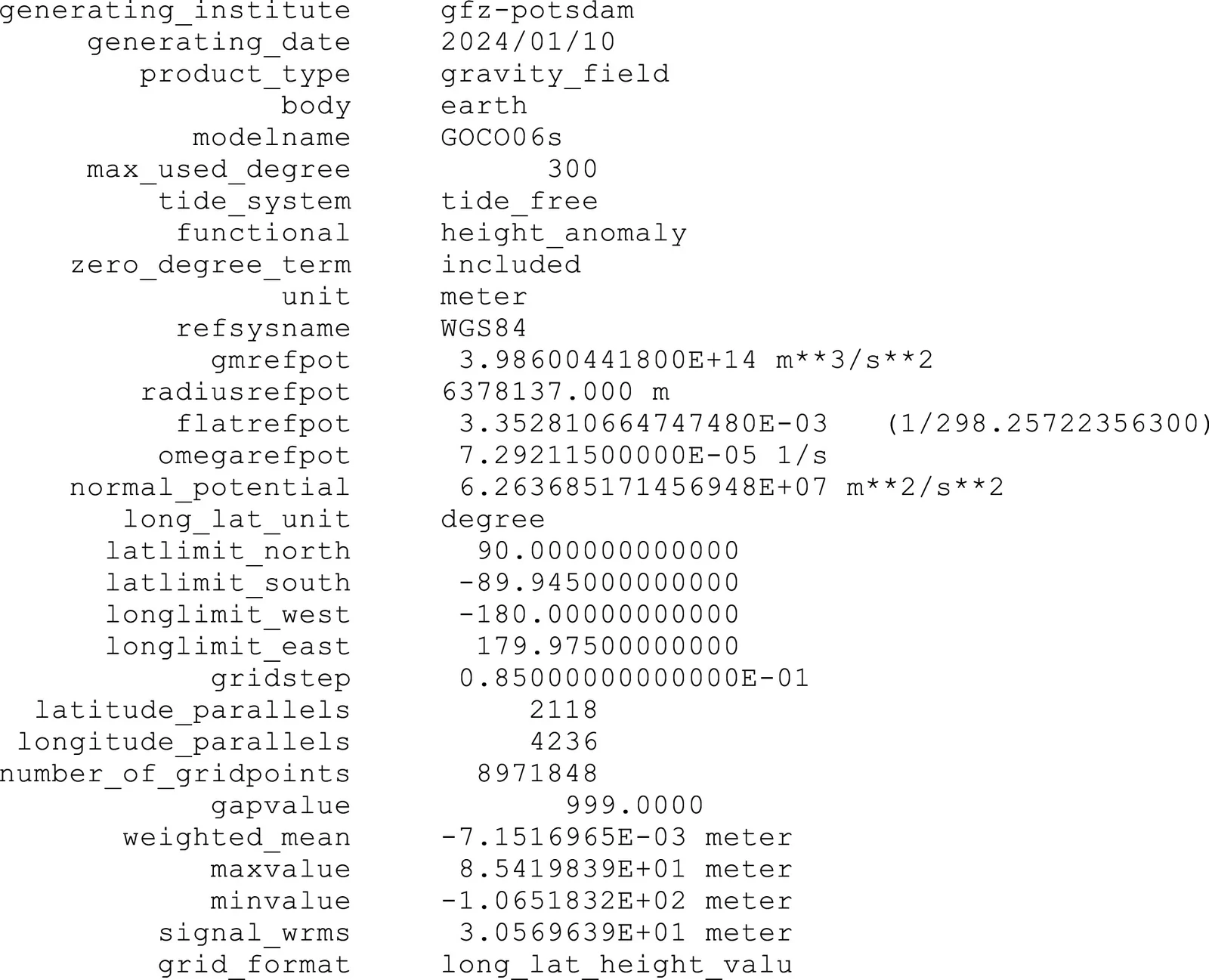
GOCO06s model configuration metadata

**Extended Data Table 4:**
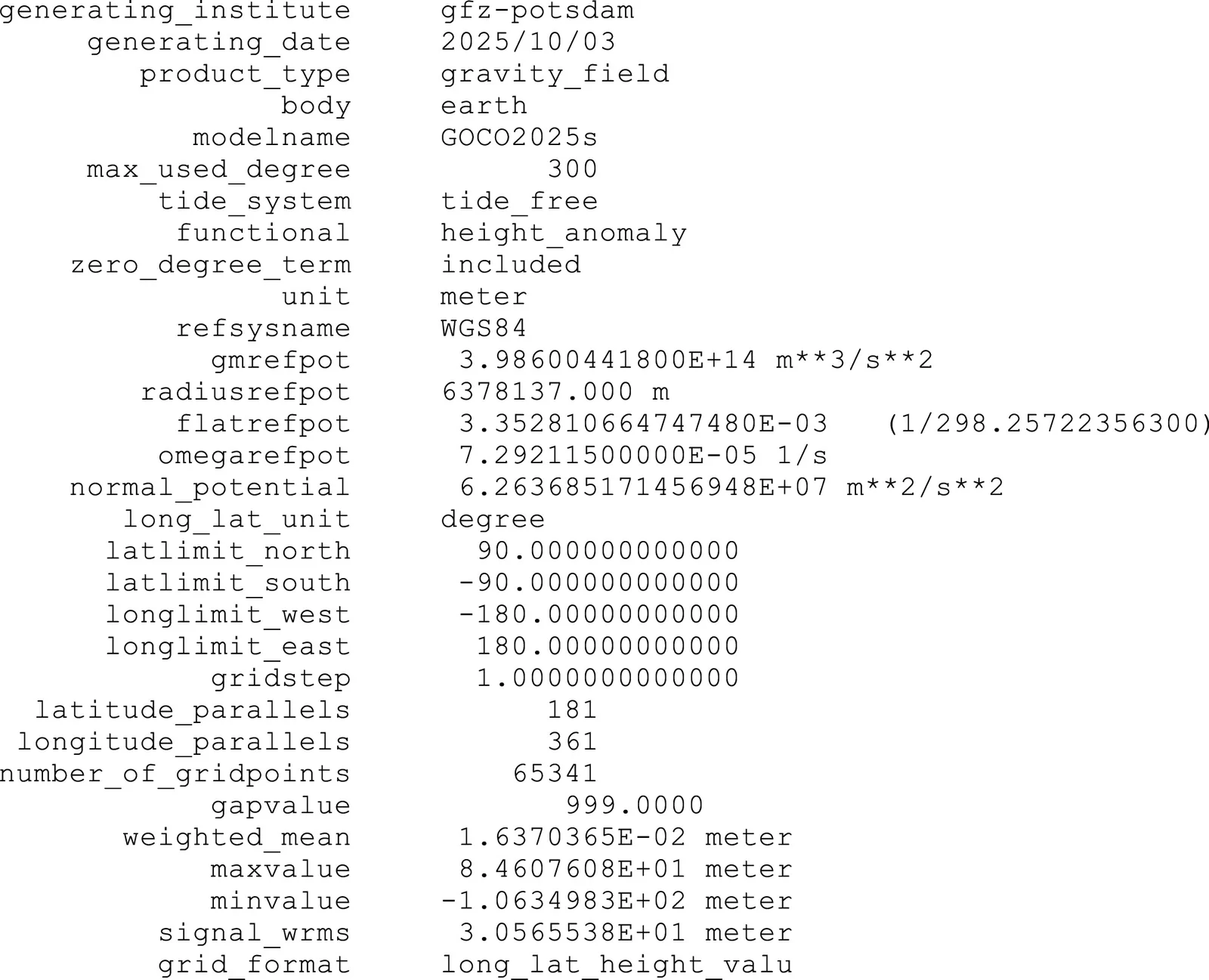
GOCO2025s model configuration metadata

**Extended Data Table 5:**
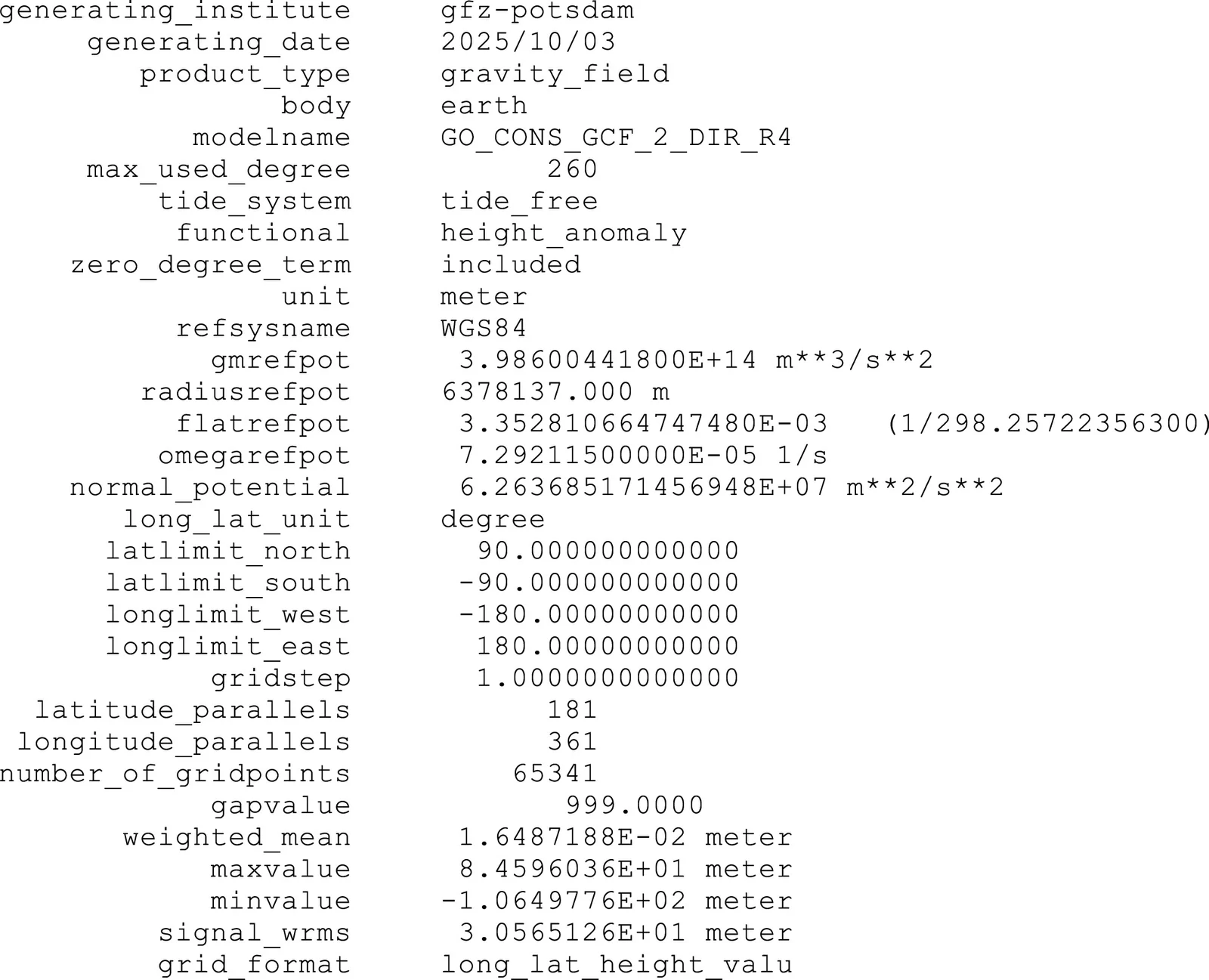
DIR R4 model configuration metadata
