# Supplementary material for: Sea level much higher than assumed in most coastal hazard assessments
Source: Nature. 2026 Mar 4;652(8110):667–74. doi: 10.1038/s41586-026-10196-1 (PMC13083249; doi:10.1038/s41586-026-10196-1)
Supplement: Supplementary file 1 — This file includes Supplementary Figs. 1–11 and Supplementary Tables 1–5 as well as the protocol for screening the evaluated literature against IPCC AR6 WG I–III and SROCC references using ChatGPT-5. [file 41586_2026_10196_MOESM1_ESM.pdf]

nature portfolio

https://doi.org/10.1038/s41586-026-10196-1

Supplementary information

Sea level much higher than assumed in most coastal hazard assessments

In the format provided by the authors and unedited

Sea level much higher than assumed in most coastal hazard assessments

Katharina Seeger[1,2,3]*, Philip S.J. Minderhoud[1,3,4]*

[1]Soil Geography and Landscape Group, Wageningen University and Research; Wageningen, The Netherlands.

[2]Institute of Geography, University of Cologne; Cologne, Germany.

[3]Department of Civil, Environmental and Architectural Engineering, University of Padova; Padova, Italy.

[4]Department of Groundwater and Water Security, Deltares Research Institute; Utrecht, The Netherlands.

*Corresponding authors. Email: Katharina Seeger (Katharina.Seeger@wur.nl, katharina.seeger@unipd.it, k.seeger@uni.koeln.de); Philip S.J. Minderhoud (Philip.Minderhoud@wur.nl)

Supplementary Information

The PDF file includes:

Supplementary Figs. 1–11

Supplementary Tables 1–5

Protocol for Screening Literature Against IPCC AR6 WG I–III and SROCC References Using ChatGPT-5

References

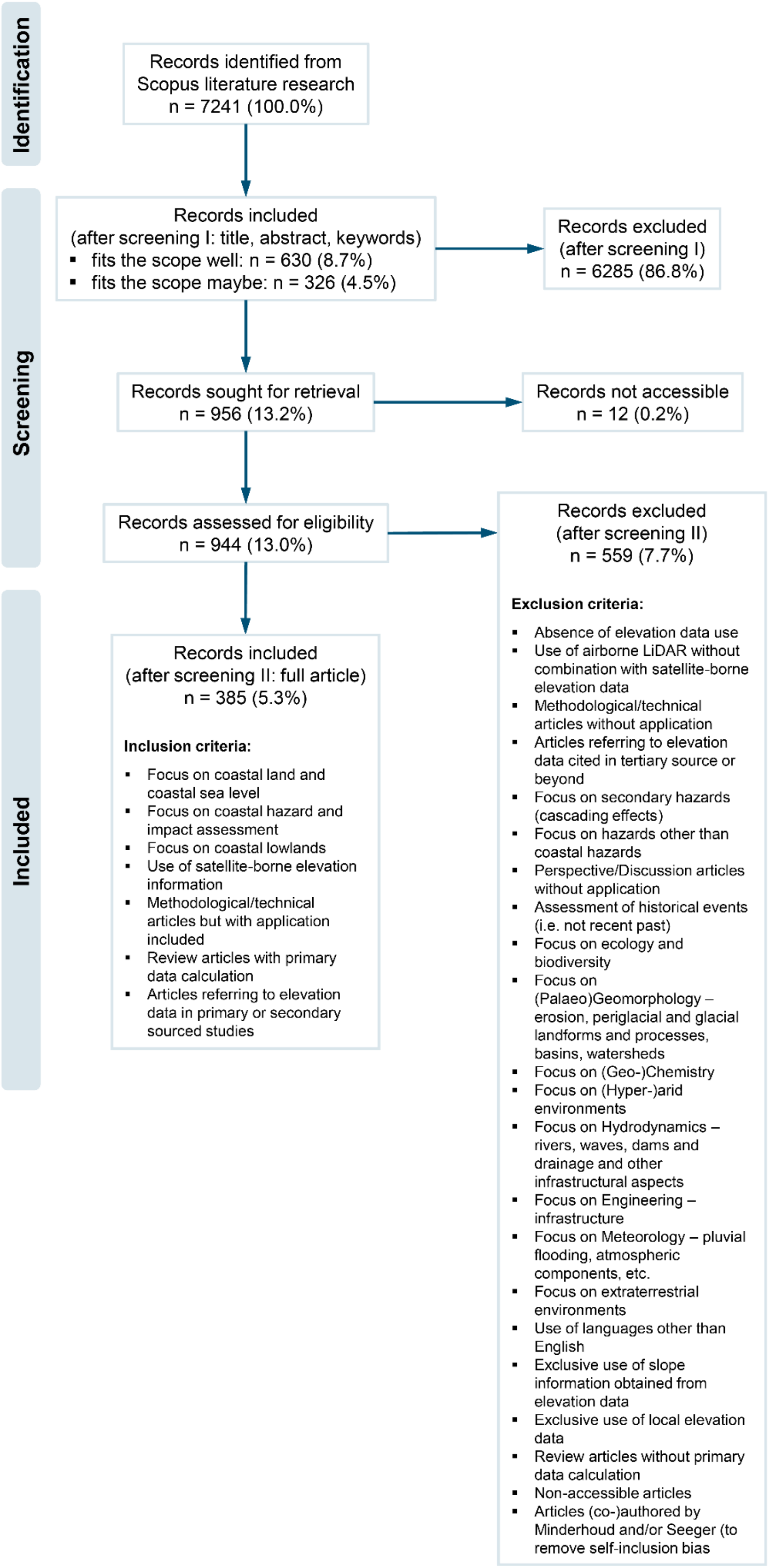

Supplementary Fig. 1 | Overview of publications searched and screened for their suitability to be included in the literature evaluation of this study. Counts per inclusion/exclusion criteria are not reported, as individual studies frequently met multiple exclusion criteria, but total inclusion and exclusion numbers are presented at each screening stage.

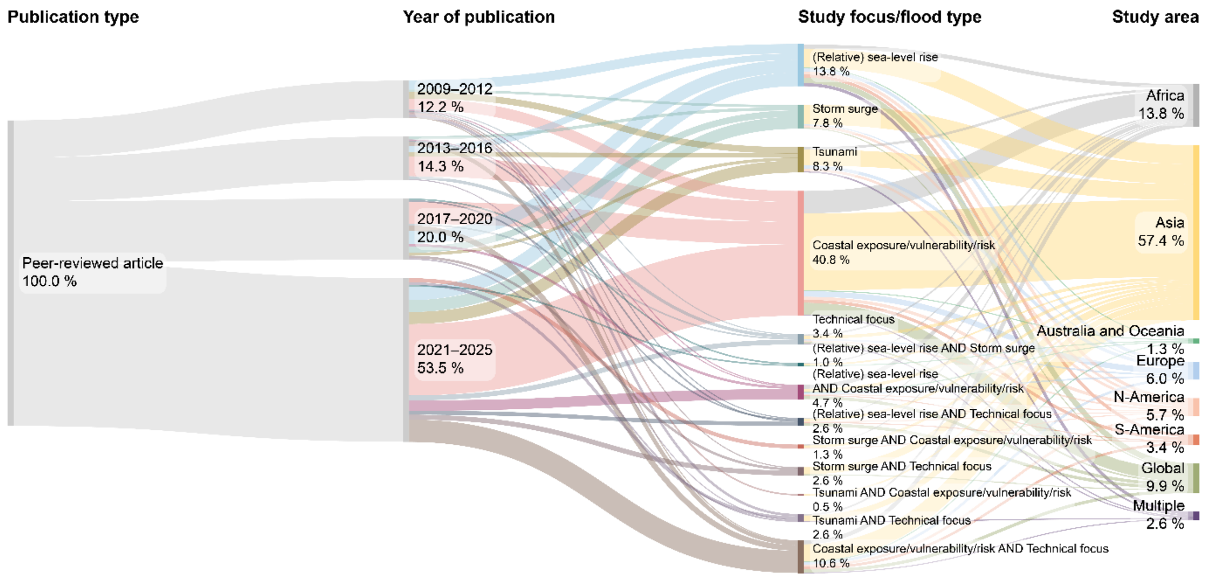

Supplementary Fig. 2 | Overview of publications included in the literature evaluation of this study. Characteristics of the literature considered in the literature evaluation of this study, including differentiation into publication type, year of publication, study focus and area of interest. The Sankey diagram for the results of this study was created using SankeyMATIC (https://sankeymatic.com/).

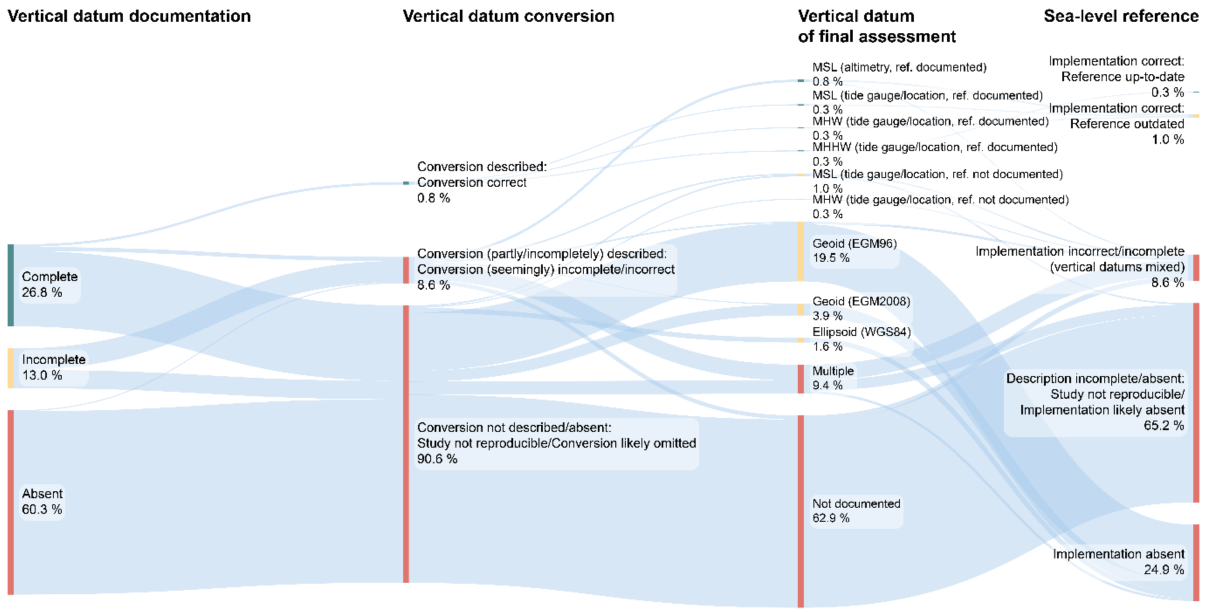

Supplementary Fig. 3 | Vertical datum types used by the literature evaluated. The Sankey diagram for the results of this study was created using SankeyMATIC (https://sankeymatic.com/).

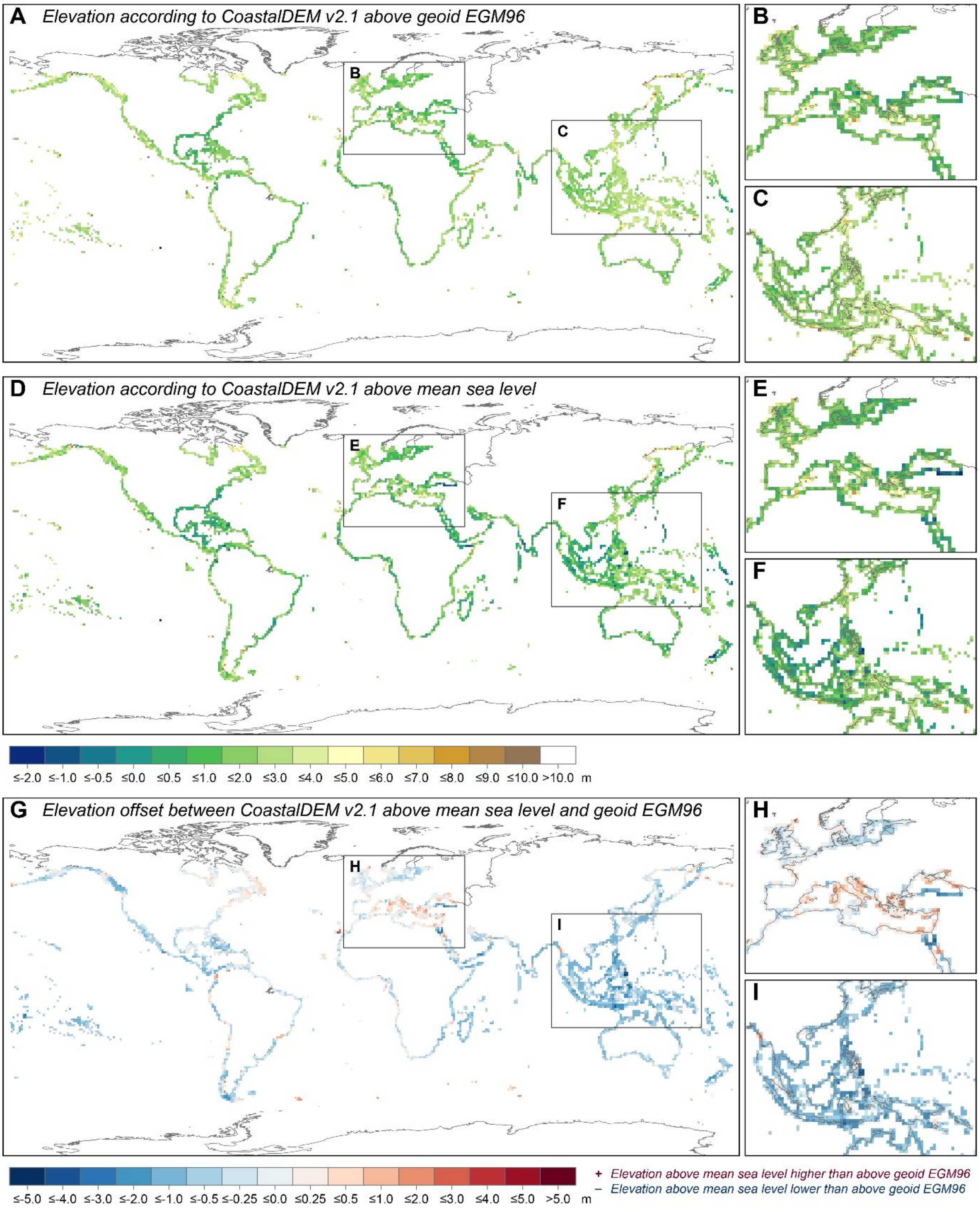

Supplementary Fig. 4 | Global coastal elevation and discrepancies between geoid-referenced and sea-level-referenced elevation. Global coastal elevation according to CoastalDEM v2.1 above (**A–C**) geoid EGM96 and (**D–F**) mean sea level (MDT HYBRID-CNES-CLS2022), as well as (**G–I**) the offset between both. For visualisation purposes, the spatial scale of the data shown was resampled to 1deg using bilinear resampling while all statistics are given at 90m spatial resolution. The results were visualised using QGIS v.3.28.6 and shapefiles from ref. [1] (Open Government Licence v3.0).

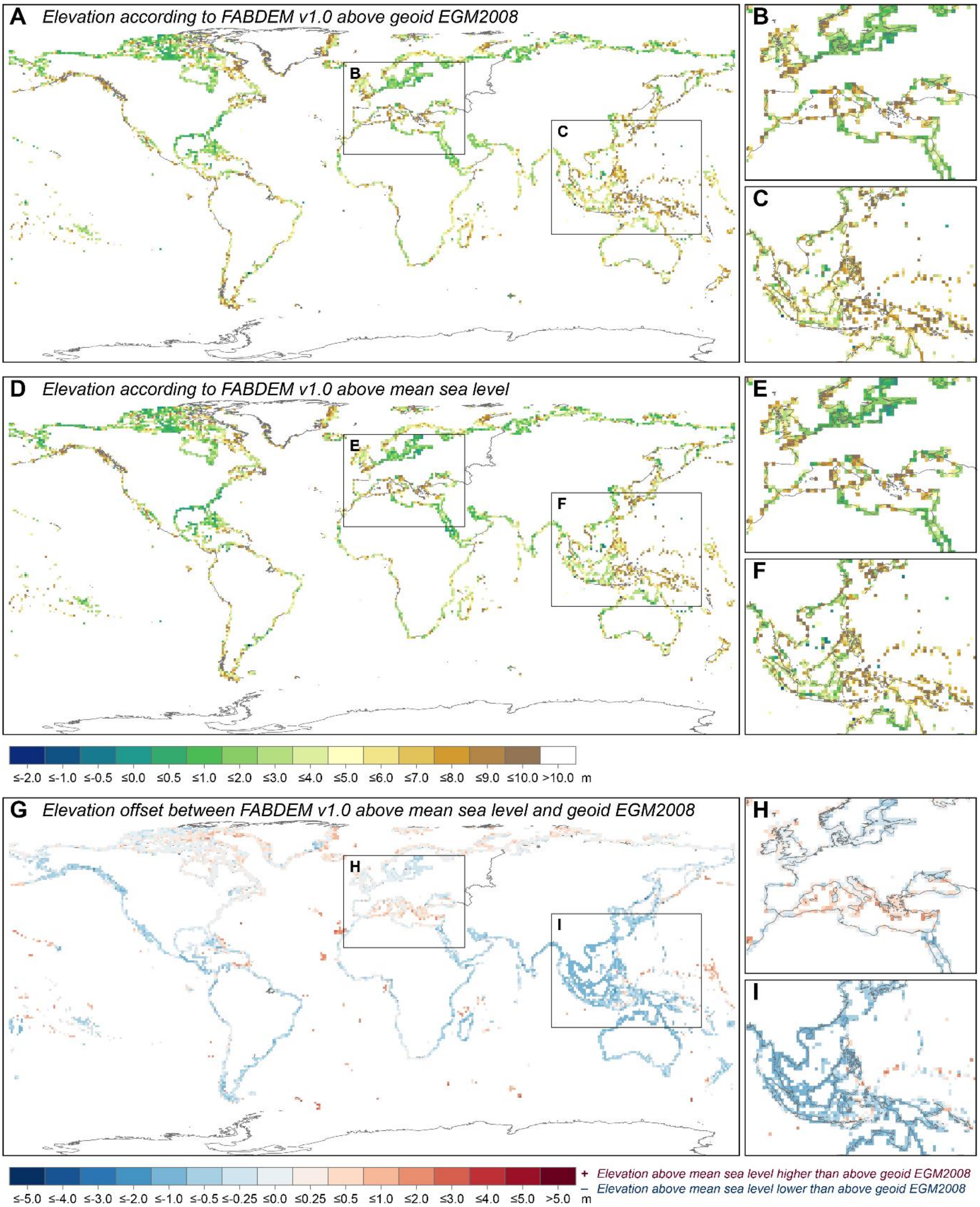

Supplementary Fig. 5 | Global coastal elevation and discrepancies between geoid-referenced and sea-level-referenced elevation. Global coastal elevation according to FABDEM v1.0 above (**A–C**) geoid EGM2008 and (**D–F**) mean sea level (MDT HYBRID-CNES-CLS2022), as well as (**G–I**) the offset between both. For visualisation purposes, the spatial scale of the data shown was resampled to 1deg using bilinear resampling while all statistics are given at 90m spatial resolution. The results were visualised using QGIS v.3.28.6 and shapefiles from ref. [1] (Open Government Licence v3.0).

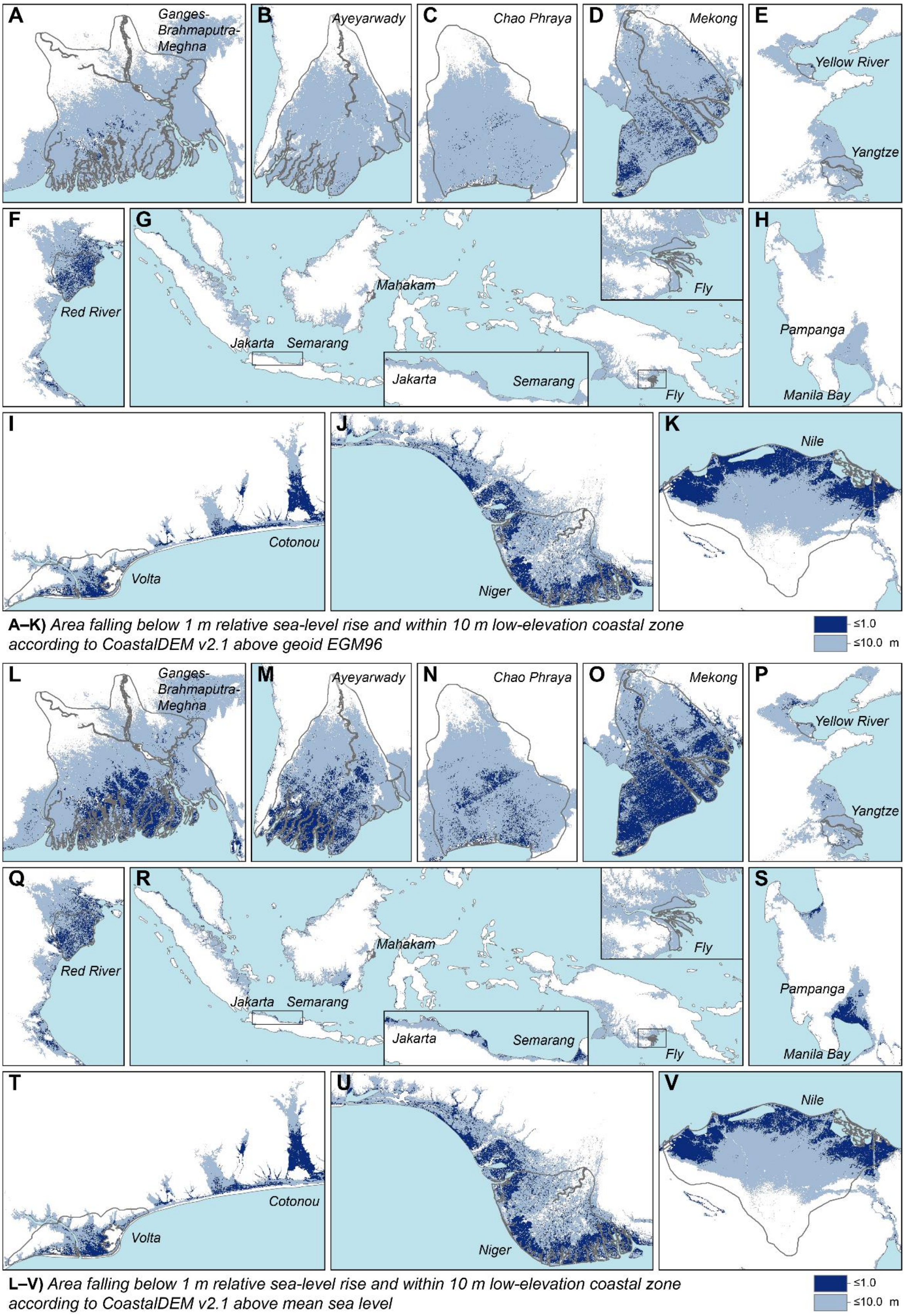

Supplementary Fig. 6 | Assessment of major low-elevated coastal-deltaic areas around the world, showing area falling below sea level by 1m RSLR and within the LECZ for geoid-referenced and sea-level-referenced elevation data. Impact of 1m RSLR and LECZ based on CoastalDEM v2.1 referenced to (**A–K**) EGM96 and (**L–V**) mean sea level (MDT HYBRID-CNES-CLS2022). The results were visualised using QGIS v.3.28.6 and shapefiles from ref. [1] (Open Government Licence v3.0) and ref. [2].

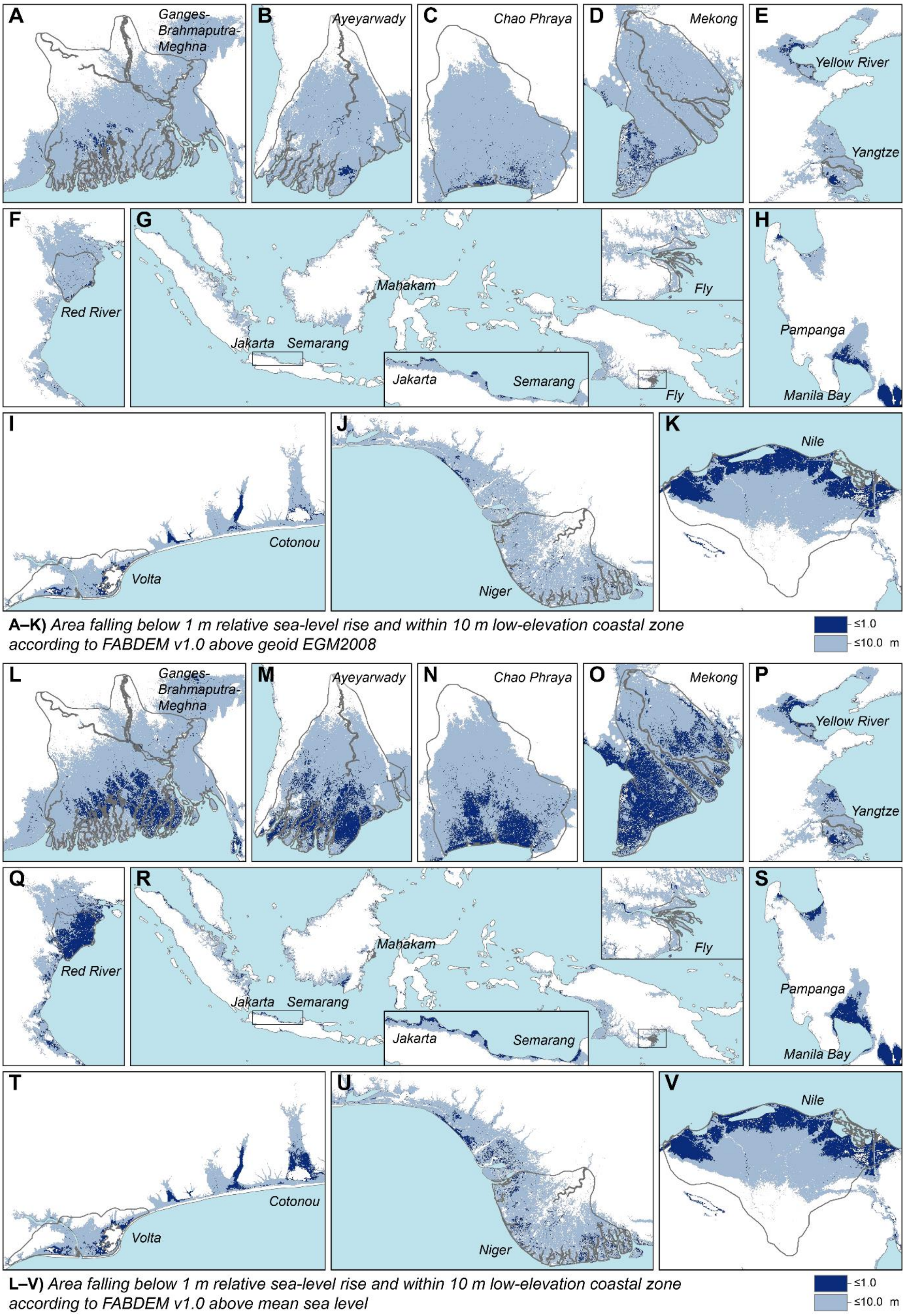

Supplementary Fig. 7 | Assessment of major low-elevated coastal-deltaic areas around the world, showing area falling below sea level by 1m RSLR and within the LECZ for geoid-referenced and sea-level-referenced elevation data. Impact of 1m RSLR and LECZ based on FABDEM v1.0 referenced to (**A–K**) EGM2008 and (**L–V**) mean sea level (MDT HYBRID-CNES-CLS2022). The results were visualised using QGIS v.3.28.6 and shapefiles from ref. [1] (Open Government Licence v3.0) and ref. [2].

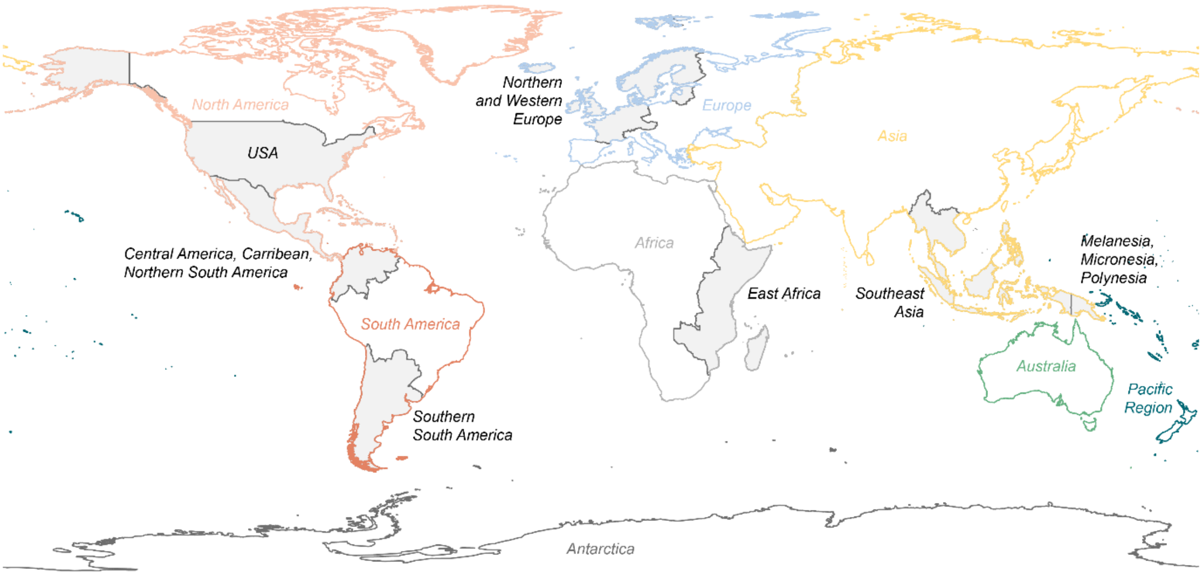

Supplementary Fig. 8 | Study areas referred to in this study. Regions and subregions used to obtain more regional statistics for assessments of coastal elevation, relative sea-level rise impact, and low-elevation coastal zone. The Pacific region excludes Papua New Guinea, the subregion Melanesia, Micronesia, Polynesia is excluding New Zealand and Hawaii). The results were visualised using QGIS v.3.28.6 and shapefiles from ref. [1] (Open Government Licence v3.0).

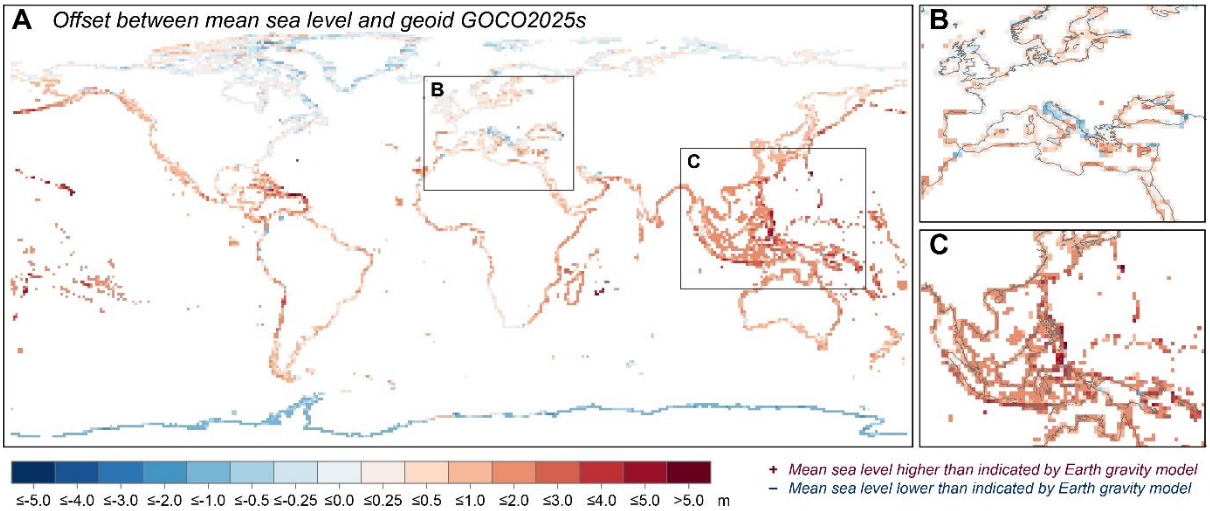

Supplementary Fig. 9 | Difference between coastal sea-level height and latest available global geoid. Actual coastal sea level as indicated by the latest available mean dynamic topography (ref. [3]) is globally underrepresented by the latest available GOCO2025s geoid (published on 11 August 2025), by on average 0.38 m (excluding Antarctica). Coastal sea level is particularly underrepresented by the geoid in more data-sparse regions, predominantly located in the Global South. This highlights the continued need for vertical datum conversion from geoid to sea level despite of improvements in global geoid modelling over time. The results were visualised using QGIS v.3.28.6 and shapefiles from ref. [1] (Open Government Licence v3.0).

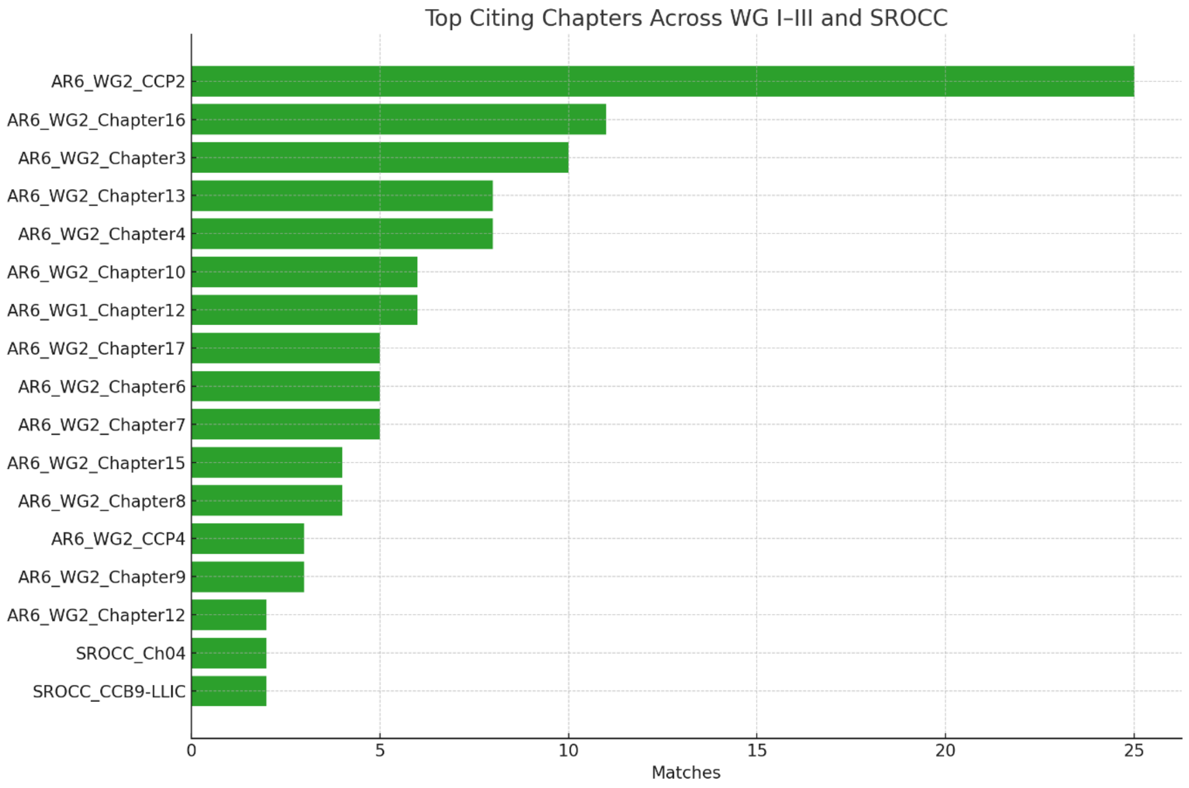

Supplementary Fig. 10 | Distribution of IPCC citations for the systematically-identified literature of this study for the entire AR6 WG1–3 and the SROCC reports. From the 385 literature references systematically identified and evaluated, 46 are included in the IPCC AR 6 WG1–3 and the SROCC reports.

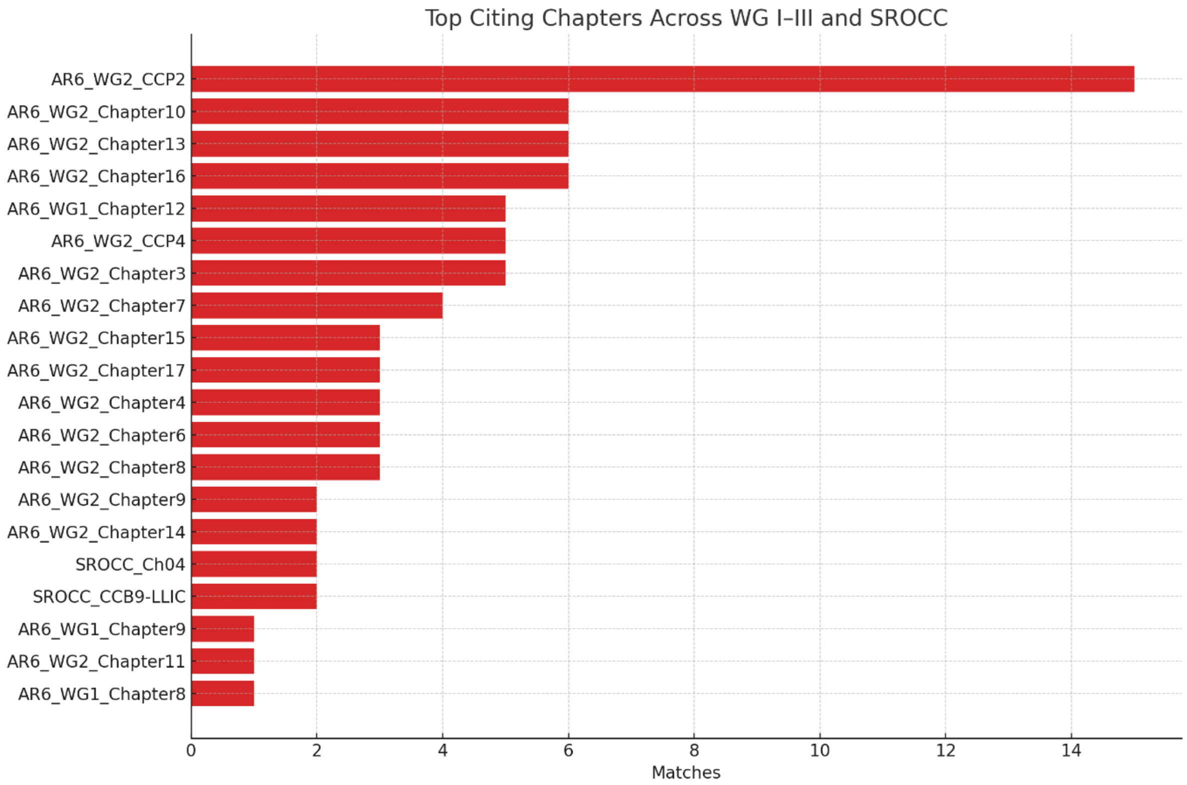

Supplementary Fig. 11 | Distribution of IPCC citations for the additionally evaluated literature of this study for the entire AR6 WG1–3 and the SROCC reports. From the 90 literature references evaluated in addition to the systematically-identified, 29 are included in the IPCC AR 6 WG1–3 and the SROCC reports.

Supplementary Table 1 | Search term and screening used to collect and select scientific literature for evaluation.

**Scopus search term**
TITLE-ABS-KEY ((delta* OR coastal-area* OR coastal-zone* OR coastal-elevation* OR coastal-lowland* OR low-elevation-coastal-zone* OR estuar* OR small-island-developing-states OR sids OR digital-elevation-model* OR dem OR digital-terrain-model* OR dtm OR digital-surface-model* OR dsm) AND (sea-level-rise OR slr OR relative-sea-level OR vertical-land-motion OR vlm OR subsidence OR inundat* OR storm-surge OR submersion OR flood* OR rising-sea*) AND (impact* OR hazard* OR assess* OR project* OR forecast* OR model* OR scenario* OR evalu* OR investig* OR future OR expos* OR risk* OR vulnera*)) AND PUBYEAR > 2008 AND PUBYEAR < 2026 AND PUBYEAR > 2008 AND PUBYEAR < 2026 AND (LIMIT-TO (DOCTYPE , "ar") OR LIMIT-TO (DOCTYPE , "re") OR LIMIT-TO (DOCTYPE , "dp")) AND (EXCLUDE (SUBJAREA , "ENER") OR EXCLUDE (SUBJAREA , "BIOC") OR EXCLUDE (SUBJAREA , "MEDI") OR EXCLUDE (SUBJAREA , "ARTS") OR EXCLUDE (SUBJAREA , "CENG") OR EXCLUDE (SUBJAREA , "CHEM") OR EXCLUDE (SUBJAREA , "IMMU") OR EXCLUDE (SUBJAREA , "PHAR") OR EXCLUDE (SUBJAREA , "VETE") OR EXCLUDE (SUBJAREA , "NEUR") OR EXCLUDE (SUBJAREA , "PSYC") OR EXCLUDE (SUBJAREA , "NURS") OR EXCLUDE (SUBJAREA , "HEAL") OR EXCLUDE (SUBJAREA , "AGRI") OR EXCLUDE (SUBJAREA , "MATE") OR EXCLUDE (SUBJAREA , "ENGI")) AND (LIMIT-TO (LANGUAGE , "English")) AND (EXCLUDE (EXACTKEYWORD , "Sediment Transport") OR EXCLUDE (EXACTKEYWORD , "Holocene") OR EXCLUDE (EXACTKEYWORD , "Seasonal Variation") OR EXCLUDE (EXACTKEYWORD , "Sedimentation") OR EXCLUDE (EXACTKEYWORD , "Sedimentology") OR EXCLUDE (EXACTKEYWORD , "Sediments") OR EXCLUDE (EXACTKEYWORD , "Stratigraphy") OR EXCLUDE (EXACTKEYWORD , "Sediment") OR EXCLUDE (EXACTKEYWORD , "Water Quality") OR EXCLUDE (EXACTKEYWORD , "River Discharge") OR EXCLUDE (EXACTKEYWORD , "Deposition") OR EXCLUDE (EXACTKEYWORD , "Depositional Environment") OR EXCLUDE (EXACTKEYWORD , "Rain") OR EXCLUDE (EXACTKEYWORD , "Runoff") OR EXCLUDE (EXACTKEYWORD , "Sequence Stratigraphy") OR EXCLUDE (EXACTKEYWORD , "Concentration (composition)") OR EXCLUDE (EXACTKEYWORD , "Paleoenvironment") OR EXCLUDE (EXACTKEYWORD , "Animals") OR EXCLUDE (EXACTKEYWORD , "Fluvial Deposit") OR EXCLUDE (EXACTKEYWORD , "Deposits") OR EXCLUDE (EXACTKEYWORD , "Rainfall") OR EXCLUDE (EXACTKEYWORD , "Drought") OR EXCLUDE (EXACTKEYWORD , "Rhizophoraceae") OR EXCLUDE (EXACTKEYWORD , "Forestry") OR EXCLUDE (EXACTKEYWORD , "Catchments") OR EXCLUDE (EXACTKEYWORD , "Ecology") OR EXCLUDE (EXACTKEYWORD , "Animal"))

**Explanation of the Scopus search term**	
Search in title, abstract and keywords	delta* OR coastal-area* OR coastal-zone* OR coastal-elevation* OR coastal-lowland* OR low-elevation-coastal-zone* OR estuar* OR small-island-developing-states OR sids OR digital-elevation-model* OR digital-terrain-model* OR dtm OR digital-surface-model* OR dsm
	**AND**
	sea-level-rise OR slr OR relative-sea-level OR vertical-land-motion OR vlm OR subsidence OR inundat* OR storm-surge OR submersion OR flood* OR rising-sea*
	**AND**
	impact* OR hazard* OR assess* OR project* OR forecast* OR model* OR scenario* OR evalu* OR investig* OR future OR expos* OR risk* OR vulnera*
	**AND**
	PUBYEAR > 2008 AND PUBYEAR < 2026

Filter search for time range of publication, document type, subject area, language and keywords	**AND**<br><br>**LIMIT-TO**<br><br>DOCTYPE<br><br>Article OR Review OR Data paper<br><br>**AND**<br><br>**EXCLUDE**<br><br>SUBJAREA<br><br>Energy OR Biochemistry, Genetics and Molecular Biology OR Medicine OR Arts and Humanities OR Chemical Engineering OR Chemistry OR Immunology and Microbiology OR Pharmacology, Toxicology and Pharmaceutics OR Veterinary OR Neuroscience OR Psychology OR Nursing OR Health Professions OR Agricultural and Biological Sciences OR Materials Science OR Engineering<br><br>**AND**<br><br>**LIMIT-TO**<br><br>LANGUAGE<br><br>English<br><br>**AND**<br><br>**EXCLUDE**<br><br>EXACTKEYWORD<br><br>Sediment Transport OR Holocene OR Seasonal Variation OR Sedimentation OR Sedimentology OR Sediments OR Stratigraphy OR Sediment OR Water Quality OR River Discharge OR Deposition OR Depositional Environment OR Rain OR Runoff OR Sequence Stratigraphy OR Concentration (composition) OR Paleoenvironment OR Animals OR Fluvial Deposit OR Deposits OR Rainfall OR Drought OR Rhizophoraceae OR Forestry OR Catchments OR Ecology OR Animal
Literature obtained<br>*(search conducted on 2025-03-27)*	*Total: n = 7241 (100.0%)*
**Literature screening**	
Criteria	INCLUSION<br><br>▪ Focus on coastal land and coastal sea level<br>▪ Focus on coastal hazard and impact assessment<br>▪ Focus on coastal lowlands<br>▪ Use of satellite-borne elevation information<br>▪ Methodological/technical articles but with application included<br>▪ Review articles with primary data calculation<br>▪ Articles referring to elevation data in primary or secondary sourced studies

	EXCLUSION<br><br>▪ Absence of elevation data use<br>▪ Use of airborne LiDAR without combination with satellite-borne elevation data<br>▪ Methodological/technical articles without application<br>▪ Articles referring to elevation data cited in tertiary source or beyond<br>▪ Focus on secondary hazards (cascading effects)<br>▪ Focus on hazards other than coastal hazards<br>▪ Perspective/Discussion articles without application<br>▪ Assessment of historical events (i.e. not recent past)<br>▪ Focus on ecology and biodiversity<br>▪ Focus on (Palaeo)Geomorphology – erosion, periglacial and glacial landforms and processes, basins, watersheds<br>▪ Focus on (Geo-)Chemistry<br>▪ Focus on (Hyper-)arid environments<br>▪ Focus on Hydrodynamics – rivers, waves, dams and drainage and other infrastructural aspects<br>▪ Focus on Engineering – infrastructure<br>▪ Focus on Meteorology – pluvial flooding, atmospheric components, etc.<br>▪ Focus on extraterrestrial environments<br>▪ Use of languages other than English<br>▪ Exclusive use of slope information obtained from elevation data<br>▪ Exclusive use of local elevation data<br>▪ Review articles without primary data calculation<br>▪ Non-accessible articles<br>▪ Articles (co-)authored by Minderhoud and/or Seeger (to remove self-inclusion bias)
Literature obtained<br>*(after screening I: title, abstract, keywords)*	*Included (fits the scope well): n = 630 (8.7%)*<br>*Included (fits the scope maybe): n = 326 (4.5%)*<br>*Excluded (does not fit the scope): n = 6285 (86.8%)*
Literature obtained<br>*(after screening II: full article)*	*Included: n = 385 (5.3%)*<br>*Excluded: n = 559 (7.7%)*<br>*Not accessible: n = 12 (0.2%)*

Supplementary Table 2 | Code description of evaluation categories used to evaluate scientific literature.

Code category	Code	Definition
Authors	------	List of authors.
**Publication type**		
Peer-reviewed article	1	Publication in an international, scientific peer-reviewed journal.
Report	2	Publication entailing systematic documentation of scientific findings, detailing practical measures and/or programs (such as development directives), or documenting technical details.
Preprint	3	Publication of a scientific manuscript prior to peer-review process.
Conference paper	4	Publication of a scientific conference contribution.
Non-peer reviewed article	5	Publication in a non-peer-reviewed journal or publication series with articles similarly structured as in peer-reviewed journals.
Book chapter	6	Publication as section in a monography or book series.
**Year of publication**		
2009	1	Publication in 2009 CE.
2010	2	Publication in 2010 CE.
2011	3	Publication in 2011 CE.
2012	4	Publication in 2012 CE.
2013	5	Publication in 2013 CE.
2014	6	Publication in 2014 CE.
2015	7	Publication in 2015 CE.
2016	8	Publication in 2016 CE.
2017	9	Publication in 2017 CE.
2018	10	Publication in 2018 CE.
2019	11	Publication in 2019 CE.
2020	12	Publication in 2020 CE.
2021	13	Publication in 2021 CE.
2022	14	Publication in 2022 CE.
2023	15	Publication in 2023 CE.
2024	16	Publication in 2024 CE.
2025	17	Publication in 2025 CE.
**Study focus/Flood type**		
(Relative) sea-level rise	1	Focus on sea-level rise or sea-level rise impact from either absolute or relative sea-level rise (i.e. with or without including vertical land motion).
Storm surge	2	Focus on storm surge hazard or storm surge impact.

Tsunami	3	Focus on tsunami hazard or tsunami impact.
Coastal exposure/vulnerability/risk	4	Focus on the assessment of coastal exposure, vulnerability, or risk, including also hazard or impact assessments without referring to specific flood types, or referring to extreme water levels resulting from multiple causes. Publications referring to assessments of low-elevation coastal zones (LECZ) belong to this category as well.
Technical focus	5	Focus on technical aspects or methodological setups/workflows in terms of data handling and/or modelling(s).
(Relative) sea-level rise AND Storm surge	6	Focus including topics of both categories *(Relative) sea-level rise* and *Storm surge*.
(Relative) sea-level rise AND Tsunami	7	Focus including topics of both categories *(Relative) sea-level rise* and *Tsunami*.
(Relative) sea-level rise AND Coastal exposure/vulnerability/risk	8	Focus including topics of both categories *(Relative) sea-level rise* and *Coastal exposure/vulnerability/risk*.
(Relative) sea-level rise AND Technical focus	9	Focus including topics of both categories *(Relative) sea-level rise* and *Technical focus*.
Storm surge AND Tsunami	10	Focus including topics of both categories *Storm surge* and *Tsunami*.
Storm surge AND Coastal exposure/vulnerability/risk	11	Focus including topics of both categories *Storm surge* and *Coastal exposure/vulnerability/risk*.
Storm surge AND Technical focus	12	Focus including topics of both categories *Storm surge* and *Technical focus*.
Tsunami AND Coastal exposure/vulnerability/risk	13	Focus including topics of both categories *Tsunami* and *Coastal exposure/vulnerability/risk*.
Tsunami AND Technical focus	14	Focus including topics of both categories *Tsunami* and *Technical focus*.
Coastal exposure/vulnerability/risk AND Technical focus	15	Focus including topics of both categories *Coastal exposure/vulnerability/risk* and *Technical focus*.
**Study area**		
Africa	1	Study area located on the continent of Africa.
Antarctica	2	Study area located on the continent of Antarctica.
Asia	3	Study area located on the continent of Asia.
Australia and Oceania	4	Study area located on the continent of Australia and Oceania.
Europe	5	Study area located on the continent of Europe.
North America	6	Study area located on the continent of North America.
South America	7	Study area located on the continent of South America.
Global	8	Study area includes the global scale.
Multiple	9	Study area includes locations on several continents.
**N DEMs**		
1	1	1 DEM considered in the publication.
2	2	2 DEMs considered in the publication.

3	3	3 DEMs considered in the publication.
4	4	4 DEMs considered in the publication.
5	5	5 DEMs considered in the publication.
6	6	6 DEMs considered in the publication.
7	7	7 DEMs considered in the publication.
8	8	8 DEMs considered in the publication.
9	9	9 DEMs considered in the publication.
10	10	10 DEMs considered in the publication.
11	11	11 DEMs considered in the publication.
12	12	12 DEMs considered in the publication.
13	13	13 DEMs considered in the publication.
14	14	14 DEMs considered in the publication.
Not known	100	Number of DEMs considered in the publication is not known/cannot be assessed.
**Actuality of DEM(s)**		
Actual	1	The evaluated publication refers to the latest available elevation dataset at the time of submission/conference (publication in case of non-peer-reviewed publication). If local elevation data was used, it was considered to be the latest available information.
Outdated	2	The evaluated publication refers to an elevation dataset that is already outdated at the time of submission/conference (publication in case of non-peer-reviewed publication).
Multiple DEMs (both actual and outdated)	3	The evaluated publication refers to min. 2 elevation datasets including both actual and outdated datasets at the time of submission/conference (publication in case of non-peer-reviewed publication). If local elevation data was used, it was considered to be the latest available information.
Multiple DEMs (actual)	4	The evaluated publication refers to min. 2 elevation datasets that are the latest available ones at the time of submission/conference (publication in case of non-peer-reviewed publication). If local elevation data was used, it was considered to be the latest available information.
Multiple DEMs (outdated)	5	The evaluated publication refers to min. 2 elevation datasets that are already outdated at the time of submission/conference (publication in case of non-peer-reviewed publication).
N/A	100	The evaluated publication does not provide any documentation or reference on the elevation data used so its actuality cannot be assessed.
**DEM accuracy assessment**		
Included	1	The evaluated publication includes an accuracy assessment of the elevation data used for the area under study.
Not included	2	The evaluated publication does not include an accuracy assessment of the elevation data used for the area under study.

**Vertical datum documentation**		
Complete	1	Documentation on the vertical datum is complete if the evaluated publication itself provides the name of the vertical datum (e.g. EGM96) or if it cites a reference that documents the name of the vertical datum of the dataset used.
Incomplete	2	Documentation on the vertical datum is incomplete if part of the vertical datum documentation is missing (e.g. information that the datum type is geoid but specific geoid not specified). This also holds true if a reference is cited where the vertical datum is not specified (i.e. specific datum name like EGM96).
Absent	3	No documentation about the vertical datum by the evaluated publication itself nor by provided references.
**Vertical datum conversion**		
Vertical datum conversion described: Conversion correct	1	All datasets used (including topography, bathymetry and sea-level datasets) are converted to the same vertical reference frame (e.g. EGM96, NAVD88) and datum conversion was conducted correctly, including all steps needed to convert all datasets used to a common vertical datum.
Vertical datum conversion (partly/incompletely) described: Conversion (seemingly) incomplete/incorrect	2	Vertical datum conversion is either incomplete (i.e. part of datum conversion is missing) or was incorrectly conducted before the conduction of the hazard assessment (e.g. elevating sea level to assess flooding or sea-level rise impacts.
Vertical datum conversion not described/absent: Study not reproducible/Conversion likely omitted	3	The publication does not provide any documentation about vertical datum conversion. This category includes also datasets that are referenced to a global vertical datum (such as global geoid or ellipsoid) and where no datum conversion was documented before conduction the hazard assessment (e.g. elevating sea level to assess flooding or sea-level rise impacts. The absence of documentation about vertical datum conversion makes the study irreproducible and we presume that vertical datum conversion was likely omitted.
N/A	4	The correctness of vertical datum conversion is not applicable as the evaluated publication by purpose included datasets referenced to different vertical datums in order to highlight the discrepancies that exist when using a local, tidal datum and a global geoid or ellipsoid.
**Vertical datum of final assessment**		
Geoid (EGM96)	1	Global EGM96 geoid.
Geoid (EGM2008)	2	Global EGM2008 geoid.
Ellipsoid (WGS84)	3	Global WGS84 ellipsoid.
Tidal (MSL, altimetry, reference documented)	4	Mean sea level estimated from altimetry data, with reference provided.
Tidal (MSL, altimetry, reference not documented)	5	Mean sea level estimated from altimetry data, no reference provided.
Tidal (MHHW, altimetry, reference documented)	6	Mean higher high water estimated from altimetry data, with reference provided.

Tidal (MHHW, altimetry, reference not documented)	7	Mean higher high water estimated from altimetry data, no reference provided.
Tidal (MSL, tide gauge or location, reference documented)	8	Mean sea level estimated from tide gauge data or location, with reference provided.
Tidal (MSL, tide gauge or location, reference not documented)	9	Mean sea level estimated from tide gauge data or location, no reference provided.
Tidal (MHW, tide gauge or location, reference documented)	10	Mean high water estimated from tide gauge data or location, with reference provided.
Tidal (MHW, tide gauge or location, reference not documented)	11	Mean high water estimated from tide gauge data or location, no reference provided.
Tidal (MHHW, tide gauge or location, reference documented)	12	Mean higher high water estimated from tide gauge data or location, with reference provided.
Tidal (MHHW, tide gauge or location, reference not documented)	13	Mean higher high water estimated from tide gauge data or location, no reference provided.
Tidal (MLLW, tide gauge or location, reference documented)	14	Mean lower low water estimated from tide gauge data or location, with reference provided.
Tidal (MLLW, tide gauge or location, reference not documented)	15	Mean lower low water estimated from tide gauge data or location, no reference provided.
Multiple	16	The datasets used are referenced to different vertical reference systems and conversion documentation is absent or incomplete. This category also includes incorrectly conducted vertical datum conversions as these include errors (i.e. artificial offsets) to the targeted vertical datum.
Not documented	17	The evaluated publication does not provide any unequivocal documentation or reference about the vertical datum type used.
**Sea-level reference**		
Implementation correct: Reference up-to-date	1	The evaluated publication refers to available sea-level data (e.g. dataset of mean sea level or high water, tidal data or datum) and is implemented correctly and datum conversion is complete<br><br>*Reference actuality:* The evaluated publication refers to the latest available sea-level data (e.g. dataset of mean sea level or high water, tidal data or datum) at the time of submission/conference (publication in case of non-peer-reviewed publication), or – in case an outdated sea-level reference was used – includes a correction for sea-level change for the time between datum establishment and time of submission/conference (publication in case of non-peer-reviewed publication). The past unavailability of nowadays available more accurate datasets was considered by evaluating whether the investigated literature used the latest available elevation and sea-level data at the time of three months before submission of the paper. In case a study uses a tide gauge or other locally measured sea-level dataset, the tidal reference of this data was correctly described and datum is actual if sea-level changes since datum establishment/time of reference water level were properly considered.

Implementation correct: Reference outdated	2	The evaluated publication refers to sea-level information that is already outdated at the time of submission/conference (publication in case of non-peer-reviewed publication), or – in case an outdated sea-level reference was used – does not include a correction for sea-level change for the time between datum establishment and time of submission/conference (publication in case of non-peer-reviewed publication).
Implementation incorrect/incomplete (vertical datums mixed)	3	The evaluated publication incorrectly or incompletely integrates different datums and/or sea-level datasets (e.g., by combining data referenced to different vertical datums (without applying a proper datum conversion), or using a specific sea-level dataset but missing documentation or part of the documentation on its implementation). This category also includes incorrectly conducted vertical datum conversions (e.g. mixing up different geoid models) as these include introducing errors (i.e. artificial offsets) to the targeted sea-level datum.
Description incomplete/absent: Study not reproducible/Implementation likely absent	4	The evaluated publication does not provide documentation or reference about the sea-level dataset(s) used or where the establishment of the sea-level datum is not specified. This category also includes evaluated publications that do not provide unequivocal information on the vertical datum type used as it remains unclear whether a sea-level datum was used.
Implementation absent	5	The evaluated publication does not apply sea-level data but refers to a global or local geoid or ellipsoid model.

Supplementary Table 3 | Quantification and attribution of uncertainties associated with global digital elevation models (DEMs) in their performance to correctly quantify the elevation of the Vietnamese Mekong Delta (ref. [4]).

a) Uncertainties related to coastal elevation assessments for mean sea level of 04/2007 (as indicated by $MDT_{1993–07/2021}$ (ref. [3]))

DEM	Inaccuracy	Extraction-induced land subsidence until 2007	Absolute sea-level change until 2007	Vertical datum offset	*Total error*
CoastalDEM v2.1	1.12 m (63.67%)	0.05 m (3.04%)	0.03 m (1.63%)	**0.56 m (31.65%)**	*1.76 m (100.00%)*
FABDEM v1.0	1.43 m (67.32%)	0.00 m (0.00%)	0.00 m (0.00%)	**0.69 m (32.68%)**	*2.12 m (100.00%)*
GLL-DTM v2	0.35 m (75.03%)	0.00 m (0.00%)	0.00 m (0.00%)	**0.12 m (24.97%)**	*0.47 m (100.00%)*
DeltaDTM v1	0.49 m (40.43%)	0.00 m (0.00%)	0.00 m (0.00%)	**0.72 m (59.57%)**	*1.21 m (100.00%)*

b) Uncertainties related to coastal elevation assessments for mean sea level of 04/2007 (as indicated by $MDT_{1993–07/2021}$ (ref. [3])) updated to 2025

DEM	Inaccuracy	Extraction-induced land subsidence until 2007	Extraction-induced land subsidence from 2008 to 2025	Absolute sea-level change until 2007	Absolute sea-level change from 2008 to 2025	Vertical datum offset	*Total error*
CoastalDEM v2.1	1.12 m (63.67%)	0.05 m (2.68%)	0.16 m (7.80%)	0.03 m (1.44%)	0.08 m (4.02%)	**0.56 m (27.91%)**	*2.00 m (100.00%)*
FABDEM v1.0	1.43 m (62.90%)	0.00 m (0.00%)	0.10 m (4.30%)	0.00 m (0.00%)	0.05 m (2.27%)	**0.69 m (30.54%)**	*2.27 m (100.00%)*
GLL-DTM v2	0.35 m (67.09%)	0.00 m (0.00%)	0.04 m (6.84%)	0.00 m (0.00%)	0.02 m (3.75%)	**0.12 m (22.33%)**	*0.52 m (100.00%)*
DeltaDTM v1	0.49 m (39.09%)	0.00 m (0.00%)	0.03 m (2.15%)	0.00 m (0.00%)	0.02 m (1.18%)	**0.72 m (57.59%)**	*1.25 m (100.00%)*

Supplementary Table 4 | Local meta-analysis and impact assessment for the Vietnamese Mekong delta. Area and population currently already below mean sea level (MSL) and falling below MSL following 1m relative sea-level rise (RSLR) for mimicked errors of incomplete vertical datum conversion and absence of vertical datum conversion in the Vietnamese Mekong Delta in absolute numbers (in km^2, population counts) and relative (in %) to total area and population of the delta, as well as their discrepancy from area and population estimates for correctly conducted assessments with vertical datum conversion to actual local MSL.

Vertical reference issue	Vertical offset with MDT (m)	Impact assessment original	Impact assessment re-evaluated	Discrepancy
**Incomplete vertical datum conversion**<br>Omitting conversion of MDT data from EGM-DIR4 to EGM96 (See Extended Data Fig. 9 for spatial global error)	*0.14 m (mean)*<br>*0.17 m (median)*<br>*0.25 m (SD.)*<br>*-0.39 m (negative max.)*<br>*0.54 m (max.)*	Area below MSL:<br>1,712 km^2 (4.46%)<br><br>Population below MSL (WorldPop 2020):<br>445,698 (2.45%)<br><br>Population below MSL (LandScan 2020):<br>263,972 (1.52%)<br><br>Population below MSL (LandScan 2023):<br>292,835 (1.61%)<br><br>Area below MSL + 1mRSLR:<br>20,858 km^2 (54.30%)<br><br>Population below MSL + 1m RSLR (WorldPop 2020):<br>7,933,353 (43.68%)<br><br>Population below MSL + 1m RSLR (LandScan 2020):<br>5,523,787 (31.86%)<br><br>Population below MSL + 1m RSLR (LandScan 2023):<br>5,759,379 (31.61%)	Area below MSL:<br>795 km^2 (2.07%)<br><br>Population below MSL (WorldPop 2020):<br>257,396 (1.42%)<br><br>Population below MSL (LandScan 2020):<br>169,025 (0.98%)<br><br>Population below MSL (LandScan 2023):<br>180,274 (0.99%)<br><br>Area below MSL + 1mRSLR:<br>25,524 km^2 (66.44%)<br><br>Population below MSL + 1m RSLR (WorldPop 2020):<br>10,230,447 (56.33%)<br><br>Population below MSL + 1m RSLR (LandScan 2020):<br>6,812,371 (39.29%)<br><br>Population below MSL + 1m RSLR (LandScan 2023):<br>7,392,668 (40.57%)	Area below MSL:<br>-917 km^2 (-2.39%)<br><br>Population below MSL (WorldPop 2020):<br>-188,302 (-1.03%)<br><br>Population below MSL (LandScan 2020):<br>-94,947 (-0.54%)<br><br>Population below MSL (LandScan 2023):<br>-112,561 (-0.62%)<br><br>Area below MSL + 1mRSLR:<br>+4,666 km^2 (+12.14%)<br><br>Population below MSL + 1m RSLR (WorldPop 2020):<br>+2,297,094 (+12.65%)<br><br>Population below MSL + 1m RSLR (LandScan 2020):<br>+1,288,584 (+7.43%)<br><br>Population below MSL + 1m RSLR (LandScan 2023):<br>+1,633,289 (+8.96%)
**Absence of vertical datum conversion**<br>Omitting vertical datum conversion, assuming MSL to equal geoid	*EGM96:*<br>*1.26 m (mean)*<br>*1.29 m (median)*<br>*0.24 m (SD)*<br>*0.77 m (min.)*<br>*1.65 m (max.)*<br><br>*EGM2008:*<br>*1.17 m (mean)*<br>*1.17 m (median)*<br>*0.11 m (SD)*	Area below MSL:<br>1,870 km^2 (4.87%) (CoastalDEM v2.1, EGM96)<br>6 km^2 (0.02%) (FABDEM v1.0, EGM2008)<br>39 km^2 (0.10%) (DeltaDTM v1, EGM2008)<br><br>Population below MSL (WorldPop 2020):<br>629,474 (3.47%) (CoastalDEM v2.1, EGM96)<br>1,627 (0.01%) (FABDEM v1.0, EGM2008)<br>10,014 (0.06%) (DeltaDTM v1, EGM2008)<br><br>Population below MSL (LandScan 2020):	Area below MSL:<br>7,755 km^2 (20.19%) (CoastalDEM v2.1)<br>2,714 km^2 (7.07%) (FABDEM v1.0)<br>2,945 km^2 (7.67%) (DeltaDTM v1)<br><br>Population below MSL (WorldPop 2020):<br>3,259,795 (17.95%) (CoastalDEM v2.1)<br>791,902 (4.36%) (FABDEM v1.0)<br>854,683 (4.71%) (DeltaDTM v1)<br><br>Population below MSL (LandScan 2020):	Area below MSL:<br>+5,885 km^2 (+15.32%) (CoastalDEM v2.1)<br>+2,708 km^2 (+7.05%) (FABDEM v1.0)<br>+2,906 km^2 (+7.57%) (DeltaDTM v1)<br><br>Population below MSL (WorldPop 2020):<br>+2,630,321 (+14.48%) (CoastalDEM v2.1)<br>+790,275 (+4.35%) (FABDEM v1.0)<br>+844,669 (+4.65%) (DeltaDTM v1)<br><br>Population below MSL (LandScan 2020):

	0.84 m (min.)<br>*1.46 m (max.)*	484,911 (2.80%) (CoastalDEM v2.1, EGM96)<br>1,554 (0.01%) (FABDEM v1.0, EGM2008)<br>8,982 (0.05%) (DeltaDTM v1, EGM2008)<br><br><u>Population below MSL (LandScan 2023):</u><br>505,199 (2.77%) (CoastalDEM v2.1, EGM96)<br>1,694 (0.01%) (FABDEM v1.0, EGM2008)<br>8,427 (0.05%) (DeltaDTM v1, EGM2008)<br><br><u>Area below MSL + 1mRSLR:</u><br>6,009 km^2 (15.64%) (CoastalDEM v2.1, EGM96)<br>2,283 km^2 (5.94%) (FABDEM v1.0, EGM2008)<br>1,372 km^2 (3.57%) (DeltaDTM v1, EGM2008)<br><br><u>Population below MSL + 1m RSLR (WorldPop 2020):</u><br>2,417,406 (13.31%) (CoastalDEM v2.1, EGM96)<br>630,515 (3.47%) (FABDEM v1.0, EGM2008)<br>371,922 (2.05%) (DeltaDTM v1, EGM2008)<br><br><u>Population below MSL + 1m RSLR (LandScan 2020):</u><br>1,944,419 (11.22%) (CoastalDEM v2.1, EGM96)<br>483,339 (2.79%) (FABDEM v1.0, EGM2008)<br>312,880 (1.81%) (DeltaDTM v1, EGM2008)<br><br><u>Population below MSL + 1m RSLR (LandScan 2023):</u><br>2,011,884 (11.04%) (CoastalDEM v2.1, EGM96)<br>526,718 (2.89%) (FABDEM v1.0, EGM2008)<br>327,820 (1.80%) (DeltaDTM v1, EGM2008)	2,578,297 (14.87%) (CoastalDEM v2.1)<br>637,408 (3.68%) (FABDEM v1.0)<br>738,826 (4.26%) (DeltaDTM v1)<br><br><u>Population below MSL (LandScan 2023):</u><br>2,711,789 (14.88%) (CoastalDEM v2.1)<br>673,945 (3.70%) (FABDEM v1.0)<br>770,951 (4.23%) (DeltaDTM v1)<br><br><u>Area below MSL + 1mRSLR:</u><br>21,238 km^2 (55.29%) (CoastalDEM v2.1)<br>18,390 km^2 (47.87%) (FABDEM v1.0)<br>24,808 km^2 (64.58%) (DeltaDTM v1)<br><br><u>Population below MSL + 1m RSLR (WorldPop 2020):</u><br>9,418,633 (51.86%) (CoastalDEM v2.1)<br>6,737,974 (37.10%) (FABDEM v1.0)<br>9,982,388 (54.97%) (DeltaDTM v1)<br><br><u>Population below MSL + 1m RSLR (LandScan 2020):</u><br>7,428,873 (42.85%) (CoastalDEM v2.1)<br>5,353,668 (30.88%) (FABDEM v1.0)<br>8,664,949 (49.98%) (DeltaDTM v1)<br><br><u>Population below MSL + 1m RSLR (LandScan 2023):</u><br>7,918,006 (43.45%) (CoastalDEM v2.1)<br>5,592,171 (30.69%) (FABDEM v1.0)<br>9,110,380 (50.00%) (DeltaDTM v1)	+2,093,386 (+12.07%) (CoastalDEM v2.1)<br>+635,854 (+3.67%) (FABDEM v1.0)<br>+729,844 (+4.21%) (DeltaDTM v1)<br><br><u>Population below MSL (LandScan 2023):</u><br>+2,206,590 (+12.11%) (CoastalDEM v2.1,)<br>+672,251 (+3.69%) (FABDEM v1.0)<br>+762,524 (+4.18%) (DeltaDTM v1)<br><br><u>Area below MSL + 1mRSLR:</u><br>+15,229 km^2 (+39.65%) (CoastalDEM v2.1)<br>+16,107 km^2 (+41.93%) (FABDEM v1.0)<br>+23,436 km^2 (+61.01%) (DeltaDTM v1)<br><br><u>Population below MSL + 1m RSLR (WorldPop 2020):</u><br>+7,001,227 (+38.55%) (CoastalDEM v2.1)<br>+6,107,459 (+33.63%) (FABDEM v1.0)<br>+9,610,466 (+52.92%) (DeltaDTM v1)<br><br><u>Population below MSL + 1m RSLR (LandScan 2020):</u><br>+5,484,454 (+31.63%) (CoastalDEM v2.1)<br>+4,870,329 (+28.09%) (FABDEM v1.0)<br>+8,352,069 (+48.17%) (DeltaDTM v1)<br><br><u>Population below MSL + 1m RSLR (LandScan 2023):</u><br>+5,906,122 (+32.41%) (CoastalDEM v2.1)<br>+5,065,453 (+27.80%) (FABDEM v1.0)<br>+8,782,560 (+48.20%) (DeltaDTM v1)

Supplementary Table 5 | Evaluation of IPCC AR6 WG I–III- and SROCC-referenced coastal assessment studies: Quantification of (potential) errors due to vertical datum offsets.

Evaluated category (Studies systematic review (n=46), additional literature (n=29)	Reference assessment in IPCC report	Specific example study details	Vertical SL offset (m)	Impact assessment original	Impact assessment re-evaluated (using identical or otherwise most comparable population data)	Discrepancy	Discrepancies between original impact assessment and meta-analysis	Comparable IPCC-included studies from systematic review	Comparable IPCC-included studies from additionally evaluated literature
Sea-level reference properly included<br>(n=1 (2))	**Kulp and Strauss, 2016**<br>(MHHW based on VDatum; Elevation based on LiDAR, SRTM, GLOBE)	Specifying vertical datum and detailing conversion of all datasets used, including integration of sea-level reference	-	-	-	-	-	-	Kulp and Strauss, 2019; Robinson et al., 2020
Sea-level reference (presumingly) incorrectly included<br>(n=9 (7))	**Hooijer and Vernimmen, 2021**<br>(MSL based on MDT_CNES_CLS13; Elevation based on GLL-DTM v1; Population based on GPWv4, reference year: 2020)	Omitting conversion of MDT data from EGM-DIR R4 to EGM96 (See Extended Data Fig. 9 for spatial global error)	Offset between EGM96 and EGM-DIR R4 (1deg spatial resolution, excl. Antarctica):<br>*0.02 m (global mean)*<br>*0.01 m (global median)*<br>*0.76 m (global SD)*<br>*-4.10 m (global negative max.)*<br>*4.30 m (global max.)*	Area below MSL:<br>131,000 km^2<br><br>*Population (as of 2020) below MSL:*<br>*35 million*<br><br>Area below MSL +1 m RSLR:<br>518,000 km^2<br><br>*Population (as of 2020) below MSL +1m RSLR:*<br>*129 million*	Area below MSL:<br>173,000 km^2 (CoastalDEM v2.1)<br>234,000 km^2 (FABDEM v1.0)<br>117,000 km^2 (GLL-DTM v2)<br>199,000 km^2 (DeltaDTM v1)<br><br>*Population below MSL (WorldPop 2020):*<br>*30 million (CoastalDEM v2.1)*<br>*35 million (FABDEM v1.0)*<br>*25 million (GLL-DTM v2)*<br>*31 million (DeltaDTM v1)*<br><br>Area below MSL +1 m RSLR:<br>460,000 km^2 (CoastalDEM v2.1)<br>624,000 km^2 (FABDEM v1.0)<br>516,000 km^2 (GLL-DTM v2)<br>670,000 km^2 (DeltaDTM v1)<br><br>*Population below MSL + 1m RSLR (WorldPop 2020):*<br>*103 million (CoastalDEM v2.1)*<br>*121 million (FABDEM v1.0)*<br>*122 million (GLL-DTM v2)*<br>*132 million (DeltaDTM v1)*	Area below MSL:<br>+42,000 km^2 (CoastalDEM v2.1)<br>+103,000 km^2 (FABDEM v1.0)<br>-14,000 km^2 (GLL-DTM v2)<br>+68,000 km^2 (DeltaDTM v1)<br><br>*Population below MSL (WorldPop 2020):*<br>*-5 million (CoastalDEM v2.1)*<br>*0 million (FABDEM v1.0)*<br>*-10 million (GLL-DTM v2)*<br>*-4 million (DeltaDTM v1)*<br><br>Area below MSL +1 m RSLR:<br>-58,000 km^2 (CoastalDEM v2.1)<br>+106,000 km^2 (FABDEM v1.0)<br>-2,000 km^2 (GLL-DTM v2)<br>+152,000 km^2 (DeltaDTM v1)<br><br>*Population below MSL + 1m RSLR (WorldPop 2020):*<br>*-26 million (CoastalDEM v2.1)*<br>*-8 million (FABDEM v1.0)*<br>*-7 million (GLL-DTM v2)*<br>*+3 million (DeltaDTM v1)*	- Meta-analysis uses latest version of MDT (i.e. MDT HYBRID-CNES-CLS2022)<br>- Meta-analysis uses latest version of GLL-DTM (i.e. v2), which is of 1deg spatial resolution compared to 0.05deg of v1<br>- Meta-analysis uses WorldPop (2020), which is of 3 arcsec spatial resolution compared to 30 arcsec of GPWv4. Analyses based on GPWv4 are therefore more prone to overestimate exposure.	Brown et al., 2018;<br>Jongman et al., 2012;<br>Kirezci et al., 2020;<br>Lincke et al., 2020;<br>Lincke and Hinkel, 2021;<br>Monioudi et al., 2018;<br>Muis et al., 2017;<br>Nicholls et al., 2021;<br>Tamura et al., 2019	Edmonds et al., 2020;<br>Eilander et al., 2020;<br>Haasnoot et al., 2021;<br>Hauer et al., 2016;<br>Muis et al., 2016;<br>Paulik et al., 2020
	**Haasnoot et al., 2021**<br>(MSL based on MDT_CNES_CLS13; Elevation based on MERIT DEM; Population based on WorldPop 2020)	Omitting conversion of MDT data from EGM-DIR R4 to EGM96 (See Extended Data Fig. 9 for spatial global error)	Offset between EGM96 and EGM-DIR R4 (1deg spatial resolution, excl. Antarctica):<br>*0.02 m (global mean)*<br>*0.01 m (global median)*<br>*0.76 m (global SD)*<br>*-4.10 m (global negative max.)*<br>*4.30 m (global max.)*	Population (as of 2020) within LECZ:<br>896 million (11%)	Population within LECZ (WorldPop 2020):<br>1,074 million (13.65%) (CoastalDEM v2.1)<br>1,012 million (12.86%) (FABDEM v1.0)<br>966 million (12.28%) (GLL-DTM v2)<br>984 million (12.51%) (DeltaDTM v1)	Population within LECZ (WorldPop 2020):<br>+178 million (+2.65%) (CoastalDEM v2.1)<br>+116 million (+1.86%) (FABDEM v1.0)<br>+70 million (+1.28%) (GLL-DTM v2)<br>+88 million (+1.51%) (DeltaDTM v1)	- Meta-analysis uses latest version of MDT (i.e. MDT HYBRID-CNES-CLS2022)<br>- Meta-analysis uses most recent DEMs<br>- Estimates from Haasnoot et al. (2021) refer to the areas of the LECZ that are hydrologically connected to the sea		

Absence of sea-level reference (n=36 (20))	**Jones and O'Neill, 2016** (EGM96; Elevation based on GMTED2010; Population based on GPWv3, reference year: 2000)	Omitting vertical datum conversion, assuming MSL to equal geoid	Offset between EGM96 and MDT (90m spatial resolution, excl. Antarctica):<br>*0.27 m (global mean)*<br>*0.19 m (global median)*<br>*0.76 m (global SD)*<br>*-5.51 m (global negative max.)*<br>*7.59 m (global max.)*	Population within LECZ (2000):<br>702.167 million<br><br>Population within LECZ (2020)*:<br>893.120 million<br><br>Population within LECZ (2100):<br>742.101 million (SSP1)<br>904.974 million (SSP2)<br>1145.946 million (SSP3)<br>492.74 million (SSP4)<br>797.805 million (SSP5)	Population within LECZ (2000) (estimated from World Pop 2020)*:<br>844.373 million (CoastalDEM v2.1)<br>795.969 million (FABDEM v1.0)<br>759.753 million (GLL-DTM v2)<br>773.886 million (DeltaDTM v1)<br><br>Population within LECZ (estimated from WorldPop 2020):<br>1,073.996 million (CoastalDEM v2.1)<br>1,012.429 million (FABDEM v1.0)<br>966.364 million (GLL-DTM v2)<br>984.341 million (DeltaDTM v1)<br><br>Population within LECZ (2100) (estimated from World Pop 2020)*:<br>1,538.147 million (CoastalDEM v2.1)<br>1,449.973 million (FABDEM v1.0)<br>1,384.000 million (GLL-DTM v2)<br>1,409.745 million (DeltaDTM v1)	Population within LECZ (2000) (estimated from World Pop 2020)*:<br>+142.206 million (CoastalDEM v2.1)<br>+93.802 million (FABDEM v1.0)<br>+57.586 million (GLL-DTM v2)<br>+71.719 million (DeltaDTM v1)<br><br>Population within LECZ (WorldPop 2020):<br>+180.876 million (CoastalDEM v2.1)<br>+119.309 million (FABDEM v1.0)<br>+73.244 million (GLL-DTM v2)<br>+91.221 million (DeltaDTM v1)<br><br>Population within LECZ (2100) (estimated from World Pop 2020):<br>+392,201 million to +1,045.407 million (CoastalDEM v2.1)<br>+304.027 million to +957.233 million (FABDEM v1.0)<br>+238.054 million to +891.26 million (GLL-DTM v2)<br>+263.799 million to +917.005 million (DeltaDTM v1)	- Meta-analysis uses latest version of MDT (i.e. MDT HYBRID-CNES-CLS2022)<br>- Meta-analysis uses most recent DEMs<br>- Meta-analysis uses WorldPop (2020), which is of 3 arcsec spatial resolution compared to 2.5 arcmin of GPWv3. Analyses based on GPWv4 are therefore more prone to overestimate exposure.<br>- Meta-analysis estimates population of 2000 by inversing average annual global population growth rate of 1.21% (2000–2020)* for population datasets of 2020 and 1.17% (2000–2023)* for population datasets of 2023<br>- Meta-analysis estimates population of 2100 by using average annual global population growth rate of 0.45% (2020–2100)* for population datasets of 2020 and 0.29% (2023–2100)* for population datasets of 2023	Avelino et al., 2018;<br>Dang et al., 2018;<br>Dasgupta et al., 2016 (EGM96);<br>Diaz, 2016;<br>Fang et al., 2016;<br>Gebremichael et al., 2018 (EGM2008);<br>Hallegatte et al., 2013;<br>Hanson et al., 2011;<br>Hereher, 2010 (EGM96);<br>Hinkel et al., 2013;<br>Joshi et al., 2016;<br>Lázár et al., 2020;<br>Lichter et al., 2011;<br>Lincke and Hinkel, 2018 (WGS84);<br>Mansur et al., 2016;<br>Martyr-Koller et al., 2021 (EGM96);<br>Mehvar et al., 2018;<br>Merkens et al., 2016;<br>Merkens et al., 2018;<br>Michael et al., 2013;<br>Mokrech et al., 2015;<br>Musa et al., 2014 (EGM96);<br>Neumann et al., 2015;<br>Reimann et al., 2018 (EGM96);<br>Roebeling et al., 2013;<br>Sajjad et al., 2018;<br>Satta et al., 2017;<br>Sciance et al., 2018 (EGM96);<br>Scussolini et al., 2017;<br>Syvitski et al., 2009 (EGM96);<br>Tessler et al., 2015;<br>Vafeidis et al., 2019 (EGM96);<br>Wang et al., 2018;<br>Wetzel et al., 2012 (EGM96);<br>Yankson et al., 2017 (EGM96);<br>Yesudian et al., 2021	Antonioli et al., 2020;<br>Barbier, 2015;<br>Bell et al., 2021;<br>Brown et al., 2016;<br>Brown et al., 2018;<br>Brown et al., 2021;<br>Elshinnawy and Almaliki, 2021;<br>Hinkel et al., 2012;<br>Hinkel et al., 2018 (EGM96);<br>Kummu et al., 2016;<br>Lin et al., 2020;<br>McEvoy et al., 2021;<br>Mussi et al., 2018;<br>Rahman et al., 2019;<br>Rohmer et al., 2021;<br>Tiggeloven et al., 2020;<br>Uddin et al., 2019;<br>Yin et al., 2020

** To estimate population in 2000 and 2100 based on WorldPop 2020, LandScan 2020 and LandScan 2023 data, we integrated rates of average annual global population growth according to world population estimates from United Nations (UN). Based on UN global population estimates of 6.14 billion (2000) and 7.79 billion (2020) and using a compound annual growth rate formula, global population between 2000 and 2020 has grown by 1.21% on average. This rate was inversed to calculate 2000 population from the WorldPop 2020 and LandScan 2020 datasets. To derive population as of 2000 from LandScan 2023 data, we considered an inverse annual population trend of 1.17%, which is based on UN population estimates for 2000 and 2023 (8.05 billion). Population as of 2100 was estimated from WorldPop 2020 and LandScan 2020 data by using an average annual population growth rate of 0.45%, considering UN's medium-variant projection starting from 7.7 billion (2020) to 10.9 billion (2100). Similarly, we applied an average annual population growth rate of 0.29% (2023–2100) for the LandScan 2023 population dataset. ChatGPT was used to derive UN estimates of global population for 2000 and 2100 as well as average annual rates of population growth.*

Protocol for Screening Literature Against IPCC AR6 WG I–III and SROCC References Using ChatGPT-5

This protocol describes the methodology that was followed to assess whether a given set of literature references (provided in Excel format) had been cited in the IPCC AR6 Working Group I–III bibliographies (.bib/.txt files) and in the IPCC SROCC report (PDF chapters). The workflow was executed using ChatGPT-5 (on 22-08-2025), which was capable of parsing, normalizing, and comparing references across these sources. The method is documented to ensure reproducibility by independent users with access to the same datasets. The full interaction with ChatGPT-5 during which this protocol developed and executed can be found here: https://chatgpt.com/share/690ddc09-58c8-8012-9cff-aaa13f73376b

The screening protocol uses the following input datasets:

- **Reference lists (Excel – with four columns providing: Authors, Year, Journal and Publication title):**

  - List of systematic reviewed references (385).

- List of additional literature references (95).

- **IPCC AR6 Working Group bibliographies:**

  - WG I, II, III chapters exported as .bib/.txt files (e.g., AR6_WG1_Chapter1.txt ... AR6_WG3_Chapter17.txt)

  - WG II cross-chapter papers (e.g., AR6_WG2_CCP1.txt)

- **SROCC chapters:**

  - PDFs of individual chapters (e.g., 03_SROCC_Ch01_FINAL.pdf ... 09_SROCC_CCB9-LLIC_FINAL.pdf)

Preprocessing

All references were normalized prior to matching:

- All text was converted to lowercase

- Punctuation and special characters were removed

- Titles were tokenized into sets of uncommon words (stopwords removed)

- Journal names were normalized (formatting stripped, abbreviations handled)

- Author surnames were extracted (focus placed on first author for matching)

- Years were converted to string for exact comparison

Screening Methodology (Balanced Workflow)

The balanced workflow was applied to maximize recall while minimizing false positives. Matching rules were as follows:

1. **Year:** Had to match exactly.

2. **Title overlap:** A minimum of 0.6 overlap of uncommon words between reference title and IPCC title was required.

3. **Journal:** Relaxed match was allowed by:
   - Direct containment (e.g., 'climate risk management' vs 'clim. risk manag.')
   - Acronym equivalence (CRM vs Climate Risk Management)
   - Minor edit distance in acronyms (≤1).

4. **Authors:** Fuzzy matching was required on the first author surname:
   - Exact match, substring match, or edit distance ≤2.

5. **SROCC PDFs:** Matches were determined based on co-occurrence of:
   - Year
   - Ordered head of the title (first ~8 words)
   - And either the normalized journal name or first author surname
   - The first page containing all criteria was recorded.

Outputs

The workflow produced the following outputs:

- **Excel match tables:**
  - Systematic_review_IPCC_matches_BALANCED.xlsx
  - Additional_literature_IPCC_matches_BALANCED.xlsx

  Each row corresponded to one reference, with columns indicating whether it was found in WG I–III and/or SROCC, and in which chapters.

- **Summary Excel workbooks:**
  - Contained overall Yes/No counts, WG vs SROCC counts, and top citing chapters.

- **Visualizations (PNG):**
  - Bar charts of top citing chapters (WG and SROCC combined and ranked).

Example Inquiries to ChatGPT-5

The following types of user queries were employed during the workflow:

- "Screen this list of references against AR6 WG I–III .bib files and SROCC PDFs."
- "Merge duplicate lines so each paper has a single row with WG and SROCC results combined."
- "Provide me with a summary table and visuals of citation occurrences per chapter."
- "Re-run the workflow for an additional reference list using the balanced approach."

Tips and Potential Pitfalls

- Journal abbreviations often differed (e.g., 'Nat Hazards Earth Syst Sci' vs 'Natural Hazards and Earth System Sciences'); relaxed matching was required.

- Titles were sometimes truncated or formatted differently; lowering the title overlap threshold improved performance.

- Author names occasionally contained typos or alternate spellings; fuzzy surname matching was critical.

- SROCC references required text extraction from PDFs; page numbers were approximate and depended on PDF text layout.

- Column naming in Excel files had to be consistent ('Authors', 'Year', 'Journal/Book/Report title', 'Publication title').

Reproducibility

By following this protocol with the specified inputs, preprocessing steps, and balanced matching workflow, an independent user with access to ChatGPT-5 could replicate the screening of external reference lists against the IPCC AR6 WG I–III bibliographies and SROCC chapters. Outputs included both detailed match tables and summarized statistics suitable for systematic review or meta-analysis.

References

1. United Nations World Food Programme. World Administrative Boundaries - Countries and Territories. Huwise Data Hub https://public.opendatasoft.com/explore/dataset/world-administrative-boundaries/export/?flg=en-us (2019); last access: 2023-06-29.
2. Tessler, Z. D., Vörösmarty, C. J., Grossberg, M., Gladkova, I., Aizenman, H., Syvitski, J. P., Foufoula-Georgiou, E. (2015). Profiling risk and sustainability in coastal deltas of the world. *Science* **349**(6248), 638–643. https://doi.org/10.1126/science.aab3574.
3. CLS, Copernicus Marine Service, 2022 Hybrid Mean Dynamic Topography CNES-CLS22-CMEMS2020, CNES (2022); https://doi.org/10.24400/527896/a01-2024.010.
4. Seeger, K. & Minderhoud, P. S. J. Elevation uncertainties in the Mekong Delta quantified using a transferable approach. *Scientific Reports* **16**, 4993. https://doi.org/10.1038/s41598-026-38315-y.
